# Highlighting fibroblast plasticity in lung fibrosis: the WI-38 cell line as a model for investigating the myofibroblast and lipofibroblast switch

**DOI:** 10.7150/thno.93519

**Published:** 2024-06-11

**Authors:** Esmeralda Vásquez-Pacheco, Manuela Marega, Arun Lingampally, Julien Fassy, Marin Truchi, Kerstin Goth, Lisa Trygub, Sara Taghizadeh, Marek Bartkuhn, Ioannis Alexopoulos, Ying Dong, Kevin Lebrigand, Andreas Gunther, Chengshui Chen, JinSan Zhang, Cho-Ming Chao, Denise Al Alam, Elie El Agha, Bernard Mari, Saverio Bellusci, Stefano Rivetti

**Affiliations:** 1 Cardio-Pulmonary Institute and Department of Pulmonary and Critical Care Medicine and Infectious Diseases, Universities of Giessen and Marburg Lung Center (UGMLC), member of the German Center for Lung Research (DZL), Justus-Liebig University Giessen, Giessen, Germany.; 2 Helios Universitätsklinikum Wuppertal-Universität Witten/Herdecke, Department of Pediatrics, Centre for Clinical and Translational Research (CCTR), Helios University Hospital Wuppertal, Witten/Herdecke University, 42283 Wuppertal, Germany.; 3 Université Côte d'Azur, CNRS UMR7275, IPMC, FHU-OncoAge, IHU RespiERA, Sophia Antipolis, Valbonne, France.; 4 Institute for Lung Health (ILH), 35392 Giessen, Germany.; 5 The Quzhou Affiliated Hospital of Wenzhou Medical University, Quzhou People's Hospital, Quzhou 324000, Zhejiang, China.; 6 Key Laboratory of Interventional Pulmonology of Zhejiang Province, Department of Pulmonary and Critical Care Medicine. The First Affiliated Hospital of Wenzhou Medical University, 325000 Wenzhou, Zhejiang, China.; 7 Department of Pediatrics, Centre for Clinical and Translational Research (CCTR), Helios University Hospital Wuppertal, Witten/Herdecke University, 42283 Wuppertal, Germany.; 8 Lundquist Research Institute, Los Angeles, California.; 9 Laboratory of Extracellular Lung Matrix Remodelling, Department of Internal Medicine, Cardio-Pulmonary Institute and Institute for Lung Health, Universities of Giessen and Marburg Lung Center (UGMLC), member of the German Center for Lung Research (DZL), Justus-Liebig University Giessen, 35392 Giessen, Germany.

**Keywords:** WI-38, Lipofibroblast, Myofibroblast, reversible switch, fibrosis, lung.

## Abstract

**Background:** Myofibroblasts (MYFs) are generally considered the principal culprits in excessive extracellular matrix deposition and scar formation in the pathogenesis of lung fibrosis. Lipofibroblasts (LIFs), on the other hand, are defined by their lipid-storing capacity and are predominantly found in the alveolar regions of the lung. They have been proposed to play a protective role in lung fibrosis. We previously reported that a LIF to MYF reversible differentiation switch occurred during fibrosis formation and resolution. In this study, we tested whether WI-38 cells, a human embryonic lung fibroblast cell line, could be used to study fibroblast differentiation towards the LIF or MYF phenotype and whether this could be relevant for idiopathic pulmonary fibrosis (IPF).

**Methods:** Using WI-38 cells, Fibroblast (FIB) to MYF differentiation was triggered using TGF-β1 treatment and FIB to LIF differentiation using Metformin treatment. We also analyzed the MYF to LIF and LIF to MYF differentiation by pre-treating the WI-38 cells with TGF-β1 or Metformin respectively. We used IF, qPCR and bulk RNA-Seq to analyze the phenotypic and transcriptomic changes in the cells. We correlated our *in vitro* transcriptome data from WI-38 cells (obtained via bulk RNA sequencing) with the transcriptomic signature of LIFs and MYFs derived from the IPF cell atlas as well as with our own single-cell transcriptomic data from IPF patients-derived lung fibroblasts (LF-IPF) cultured *in vitro*. We also carried out alveolosphere assays to evaluate the ability of the proposed LIF and MYF cells to support the growth of alveolar epithelial type 2 cells.

**Results**: WI-38 cells and LF-IPF display similar phenotypical and gene expression responses to TGF-β1 and Metformin treatment. Bulk RNA-Seq analysis of WI-38 cells and LF-IPF treated with TGF-β1, or Metformin indicate similar transcriptomic changes. We also show the partial conservation of the LIF and MYF signature extracted from the Habermann *et al.* scRNA-seq dataset in WI-38 cells treated with Metformin or TGF-β1, respectively. Alveolosphere assays indicate that LIFs enhance organoid growth, while MYFs inhibit organoid growth. Finally, we provide evidence supporting the MYF to LIF and LIF to MYF reversible switch using WI-38 cells.

**Conclusions:** WI-38 cells represent a versatile and reliable model to study the intricate dynamics of fibroblast differentiation towards the MYF or LIF phenotype associated with lung fibrosis formation and resolution, providing valuable insights to drive future research.

## Introduction

Idiopathic pulmonary fibrosis (IPF) is a debilitating lung disease characterized by excessive tissue scarring, leading to compromised lung function and respiratory failure. Fibrosis disrupts the regular tissue architecture, replacing the normal lung parenchyma with a dense collagen-rich extracellular matrix (ECM) network 1.

Fibroblasts are pivotal players in the intricate web of lung fibrosis pathogenesis. These cells, often described as the architects of the ECM, are indispensable for the structural integrity of tissues. The ECM, a complex network of proteins and carbohydrates, provides mechanical support and signaling cues to cells. When fibroblasts function abnormally or become hyperactive, they can excessively produce ECM components, leading to tissue scarring observed in fibrotic diseases 2.

A particularly intriguing aspect of fibroblasts in the context of lung fibrosis is their plasticity. Under certain conditions, such as tissue injury or specific signaling cues, fibroblasts can undergo differentiation. One well-documented path is their transformation into myofibroblasts (MYFs) 3,4. Dysregulation of signaling pathways in fibroblasts, driven by factors such as TGF-β1 and others, can trigger pathological fibroblast phenotypes contributing to fibrotic tissue remodeling 5,6.

MYFs are characterized by alpha-smooth muscle actin (ACTA2; α-SMA) 7 expression and robust collagen production. They are often considered the culprits behind the excessive ECM deposition in fibrotic diseases. Their contractile properties, combined with their prolific collagen production, contribute to the stiffening of tissues, a defining feature of fibrotic lesions 8.

On the other hand, fibroblasts can also differentiate into lipofibroblasts (LIFs), a less studied but equally intriguing cell type. LIFs are recognized by their lipid droplet inclusions and have traditionally been associated with roles in lipid storage and metabolism 9. However, recent research has shed light on their multifaceted functions, especially in lung fibrosis 4. These cells are not merely passive lipid storages: they actively participate in tissue repair processes and have been implicated in the modulation of fibrosis. Their interactions with other cell types, such as alveolar type 2 cells (AT2), underscore their importance in maintaining lung homeostasis. As our understanding of LIFs deepens, it becomes evident that they play a dual role. While they can be protective and reparative under certain conditions, their dysregulation can also contribute to fibrotic progression 10.

Therefore, fibroblasts, with their potential to differentiate into either MYFs or LIFs, occupy a central position in the narrative of lung fibrosis. Deciphering the signals that dictate their fate and function is crucial for developing therapeutic strategies to halt or even reverse fibrotic diseases 11.

Yet, our mechanistic understanding of the regulation of the MYF or LIF differentiation remains incomplete, mainly due to the limited study models available.

Traditionally, primary lung fibroblast cultures derived from healthy donors and/or IPF patients have been a standard approach to studying fibroblast biology. However, the heterogeneous nature of primary cells, including growth and gene expression variability, presents challenges.

In this study, we propose the use of WI-38 cells, a human embryonic lung fibroblast cell line, as a powerful tool to study fibroblast biology and the transition between lipofibroblasts and myofibroblasts in the context of lung fibrosis. Other human embryonic cell lines were also available such as MRC5 and IMR-90. The comparison of WI-38, IMR-90 and MRC5 cells was previously reported 12. The use of IMR-90 cells is limited to passage 20 while the MRC5 and WI-38 cells can be used until passage 36. In this study, we selected the WI-38 cells because we previously reported that they represent a good model to study fibroblasts to LIF differentiation 13.

WI-38 cells have been extensively used in vaccine production 14 and studies related to aging including replicative senescence 15, cancer, and other diseases 16. They constitute a standard cell line model for cytotoxicity testing 17, due to their genetic stability, ease of handling, and reproducibility of results. However, so far, few studies focused on their differentiation potential 18,19. Their applicability as a model to study fibroblast differentiation in lung fibrosis has not been fully explored.

First, we compared the results obtained with WI-38 cells with the current primary IPF models. We induced myofibroblast differentiation using TGF-β1 treatment and lipofibroblast differentiation using Metformin treatment. We analyzed the respective phenotypical and transcriptomic changes in WI-38 cells based on the known markers for myofibroblasts and lipofibroblasts. We also compared their transcriptomic response to the ones elicited by TGF-β1 and Metformin treatment of primary fibroblasts from IPF lungs. We also tested their ability to support alveolar epithelial type 2 cell growth using alveolosphere assays. Moreover, we correlated our *in vitro* transcriptome data from WI-38 cells (obtained via bulk RNA sequencing) with the genetic signature of lipofibroblasts and myofibroblasts derived from the IPF Cell Atlas (http://ipfcellatlas.com/) 20,21 as well as with from our own single-cell transcriptomic data generated on LF-IPF.

The results of our study underscore the potential of WI-38 cells as a versatile and reliable model to study the intricate dynamics of fibroblast differentiation in lung fibrosis, providing valuable insights to drive future research in the field.

## Materials and methods

### Human-derived specimens and cell line

Human primary lung fibroblasts from IPF lungs (referred to as LF-IPF) were obtained from the European IPF registry (eurIPFreg) at the Universities of Giessen and Marburg Lung Center, which is a part of the German Center for Lung Research 22. Written consent was obtained from each patient, and the ethics committee of Justus-Liebig University Giessen approved the study. The WI-38 cell line (#CCL-75) was purchased from the American Type Culture Collection (ATCC). Cells were amplified, and frozen stocks were made. The cells were used from passage 3 through 7 for the functional assays. Cells were grown at confluence in T75 flasks (SARSTEDT cat nr 83.3911.302) and split 1:3. Three to four days are required to regain confluency.

### Cell culture

Primary lung fibroblasts derived from 4 IPF patients (LF-IPFs) and the WI-38 cell line were maintained in Dulbecco's modified Eagle's medium (DMEM) (Gibco™, cat. nr 21885-025) supplemented with 10% Fetal Bovine Serum (FBS, Gibco™, cat. nr 10270-106) and 1% penicillin/streptomycin (Gibco™, cat. nr 15140-22) at 37 °C and 5% CO_2_. Cells between passages 3 and 7 were used for the experiments. For each experiment, 3 × 10^5^ cells were seeded per well in 6-well plates (SARSTEDT cat nr 83.3920.300). The following day, the cells were starved (0% serum) for 24 h and then treated for 96 h with 5 mM Metformin (Merck, cat. nr M0605000) or 2 ng/ml TGF-β1 (Peprotech cat nr 10021). As Controls (Vehicle), the same volume of the TGF-β1 solvent (10 mM citric acid, pH 3.0 containing 0.1% bovine serum albumin) was used. For the transdifferentiation of MYF to LIF, WI-38 cells were first treated with TGF-β1 as previously mentioned, after the differentiation to MYF, the treatment was changed to 5 mM metformin for 96 h. For the transdifferentiation of LIF to MYF, WI-38 cells were first treated with metformin as previously mentioned, after the differentiation to LIF, the treatment was changed to 2 ng/ml TGF-β1 for 96 h.

### Immunofluorescence

The cells were fixed in 4% of PFA during 10 min, the cells were blocked for 1 h (3% of BSA, 0.4% Triton X in PBS 1X) at room temperature, then they were incubated with FITC-conjugated mouse monoclonal anti-ACTA2 (Sigma-Aldrich, cat. Nr. F3777) 1:200 during 1 h at room temperature or MYH11 (Prestige Antibodies power by Atlas Antibodies, Sigma Aldrich, cat. nr HPA015310) overnight at 4 °C. The following day, for MYH11 immunostaining, the cells were washed with PBS 1X and incubated during 1 h at room temperature with Alexa Fluor^TM^ 594 donkey anti-rabbit IgG (H+L) (Sigma-Aldrich, cat. nr A21207) secondary antibody 1:500 was used. After washing, the staining was mounted with DAPI (ProLong™ Gold antifade reagent with DAPI, Invitrogen^TM^ by Thermo Fisher Scientific, cat. nr P36935), and the cells were examined and imaged by fluorescence microscopy (Leica DM5500 B Automated Upright Microscope System). To analyze the presence of lipid droplets, as evidence of differentiation of the cells to a lipofibroblast phenotype, LipidTOX^TM^ green or red neutral lipid dye (Invitrogen^TM^ by Thermo Fisher Scientific, cat. nr H34475) was used (1:200) and cells when alive were placed in the incubation chamber (37 °C and 5% CO_2_) during 20 min or 20 min room temperature when the cells were fixed.

### RNA extraction and qPCR

The cells were collected in a lysis buffer according to the manufacturer's protocol, and RNA extraction was performed using the QIAcube® Connect (Qiagen). After quantification using a NanoDrop™ Spectrophotometer 2000/2000c (Thermo Fisher), the RNA was reverse transcribed using the QuantiTect Reverse Transcription kit (Qiagen, cat. nr 205314) and then diluted to a final concentration of 5 ng/µL. For qPCR, PowerUp™ Sybr Green Master mix (Applied Biosystems^TM^ by Thermo Fisher Scientific, cat. nr A25742) was used. The primer sequences are listed in Table 1.

### RNA sequencing and bioinformatic analysis

RNA-seq data of WI-38 cells were analyzed by the genomic facility of the Institute for Lung Health (ILH) at the Justus-Liebig University Giessen, Germany. For genome-wide analysis of gene expression, RNA sequencing libraries from isolated mRNA were generated and sequenced by the Institute for Lung Health (ILH) - Genomics and Bioinformatics - at the Justus-Liebig-University (JLU) Giessen (Germany). A total amount of 500 ng of RNA per sample was used to enrich for polyadenylated mRNA followed by cDNA sequencing library preparation utilizing the NEBNext® Ultra™ II Directional RNA Library Prep Kit for Illumina® according to the manufacturer's instructions. After library quality control by capillary electrophoresis (4200 TapeStation, Agilent), cDNA libraries were sequenced on the Illumina NovaSeq 6000 platform generating 50 bp paired-end reads. For demultiplexing and the subsequent FASTQ file generation we used Illumina's bcl2fastq (2.19.0.316). Primary processing of the sequencing reads, i.e., quality control, filtering, trimming, read alignment and generation of gene specific count tables was performed using the nf-core 23,24. RNA-seq v3.7 bioinformatics pipeline (NEXTFLOW version 23.04.03). The homo sapiens hg38 genome and gene annotation was used as downloaded from Illumina's iGenome repository. The pipeline run was performed with standard parameters in docker mode. The resulting tables with raw read counts were imported into R where all down-stream processing was performed. Normalization of read counts and detection of differentially expressed genes were performed using DESeq2 25. Volcano plots were generated using the EnhancedVolcano R package. For comparison of WI-38 data with publicly available scRNA-seq data, the file GSE135893_ILD_annotated_fullsize.rds.gz was downloaded from GEO entry GSE135893. Differential gene expression analysis was done using Seurat 26 function *findMarkers* in order to derive genes specifically expressed in *PLIN2*+ fibroblasts over fibroblasts and myofibroblasts over fibroblasts, using provided annotation from original publication 20. These gene signatures were subsequently compared to the WI-38 by gene set enrichment analysis using the *GSEA*-function of the clusterProfiler package 27 and visualized by the complexHeatmap package 28.

RNA-seq data has been deposited at NCBI's gene expression omnibus (GEO) under accession GSE264038.

### Cell viability

Cell viability was evaluated at 2, 24, 48, 72 and 96 h using the AlamarBlue^TM^ Cell Viability reagent (Invitrogen^TM^ by Thermo Fisher Scientific, cat nr. DAL1025), following the manufacturer's protocol. After 96 h of treatment, 3000 cells per well were seeded in 96 well plates (Greiner BIO-ONE cat nr. 655 180). AlamarBlue^TM^ Cell Viability reagent (10%) was added to each well. The viability was evaluated by subtracting the average 600 nm absorbance values of the cell culture medium alone (used as the blank reference) from the 570 nm absorbance values of experimental wells. The absorbance was measured in the Tecan InfiniteM200 plate reader.

### Scratch test for cell migration

WI-38 cells were seeded into 6 well plates at a density of 1.5 × 10^5^ cells per well for each condition previously described. The next day, after 24 h of starvation (serum-free DMEM media), the cells were treated as previously described. After 96 h of treatment, the bottom of each dish was scratched using a sterile pipette tip. After the scratch, the dishes were gently washed with PBS 1X to remove cell debris. After 24 h, the washing step was repeated. The cells were maintained in serum-free DMEM media at 37 °C and 5% CO_2_ for 96 h for the entire assay duration. Images were acquired at 0, 24, 48 and 96 h. In every condition, the measurements of the scratched area at the different time points were calibrated with the original surface of the scratched area, allowing the comparison between the different conditions. The percentage of wound closure was measured using Image J software with the wound healing size tool 29.

### FACS preparation

B6N.Cg-Tg(Sftpc,-EGFP)1Dobb/J mice (Jackson Lab, strain 028356) were euthanized. The lungs were perfused with 10 mL of PBS 1X through the heart's right ventricle. For the single-cell preparation, lungs were inflated intratracheally with dispase (5 U/mL; Corning, cat. nr 354235) and further digested by incubation with 3 mL dispase solution (5 U/mL) for 20 min at room temperature. Lungs were finely chopped and digested in collagenase type IV (Gibco, cat. nr 17104-019) for 30 min at 37 °C. The cell suspension was passed through 70 and 40 µm cell strainers (Greiner BIO-ONE, Easystrainer cat. nr 542070 and 542040, respectively). After washing, the cell suspension was stained with SYTOX^TM^ Red (Invitrogen^TM^ by Thermo Fisher Scientific, cat. nr S34859), and sorting of Sftpc-EGFP^+^ cells was carried out using the BD FACSMelody™ cytometer. Data were analyzed using FlowJo software.

### Alveolosphere assay

21000 sorted Sftpc-EGFP^+^ cells from the adult lungs of B6N.Cg-Tg(Sftpc,-EGFP)1Dobb/J mice and 20000 fibroblast, lipofibroblast or myofibroblasts were resuspended in 50 µl of organoid medium (DMEM + 10% FBS + 1% Pen/Streptomycin + ITS 1% (Gibco™, cat. nr 41400-045) + Heparin 0.1% (STEMCELL cat. nr 07980)) plus 50 µl of Matrigel (Corning, cat. nr 356231). For each insert, 100 µl of the mix was added on top of a 0.4 µm insert (Greiner BIO-ONE cat. nr 662641), placed in a 24-well plate and incubated for 7 min at 37 °C. After incubation, 500 µl of organoid medium was placed in the lower chamber, and the plate was placed for 14 days in 5% CO_2_ at 37 °C. The organoid medium was changed every second day. ROCK inhibitor (10 μM, Y27632 STEMCELL cat. nr 72304) was included in the organoid medium for the first 2 days of culture. The percentage of colony-forming efficiency (%CFE) was calculated as the ratio between the number of spheres observed over the initial number of AT2 cells multiplied by 100.

### LF-IPF stimulation and preparation for chromium^TM^ single-cell RNA-seq

LF-IPF (IPF#5 and IPF#6) were seeded at 300,000 cells/well in DMEM 10% FBS for 24 h. The following day, the cells were starved (0% serum) for 24 h and then treated or not for 72 h with 5 mM Metformin. Following trypsinization, cells from each condition were labelled with in-house Hashtag oligonucleotide (HTO)-coupled antibody (anti-CD90, BD Biosciences, Ref #550402) following the procedure of Stoeckius *et al.* 30 using the LYNX Rapid Streptavidin Antibody Conjugation Kit (Biorad, Ref #LNK163STR). Briefly, for each condition, 1x10^6^ cells were resuspended in PBS 2% BSA, 0.01% Tween and incubated with 10 μL Fc Blocking reagent for 10 min at 4 °C then stained with 0.5 μg of cell hashing antibody for 20 min at 4 °C. After washing with PBS, 2% BSA, 0.01% Tween, samples were counted and assessed for single cell separation and overall cell viability ( > 90%). Samples were then adjusted to the same concentration, mixed in PBS supplemented with 0.04% of bovine serum albumin at a final concentration of 100 cells/μl and pooled sample was immediately loaded onto 10X Genomics Chromium device to perform the single cell capture (3000 cells / condition for a total of 12,000 cells).

### Single-cell RNA-seq data processing

Libraries were prepared as recommended, following the Chromium Next GEM Single Cell 3' Reagent Feature Barcoding kit (10X Genomics). Libraries were then quantified, pooled and sequenced on an Illumina NextSeq 500. Alignment of reads from the single cell RNA-seq library and unique molecular identifiers (UMIs) counting were performed with 10X Genomics Cell Ranger tool (v3.0.2). Reads of HTOs used for Cell Hashing (BC1-4, see Table 2) were counted with CITE-seq-Count (v1.4.2). Counts matrices of total UMI and HTOs were integrated on a single object using Seurat R package, from which the data were processed for analysis. HTOs were demultiplexed with HTODemux in order to assign LF-IPF identity and treatment. Only cells identified as “Singlet” after both demultiplexing and passing quality control thresholds of UMI and mitochondrial content were kept. Differential expression analyses between cells from control and metformin treated LF-IPF were carried out with DESeq2 (v1.30.1). Genes considered as differentially expressed were selected according to an adjusted p_value_ threshold of 0.05, obtained with the Wald test and the Benjamini-Hochberg method for multiple tests correction implemented in DESeq2.

### Statistical analysis

Statistical analyses and graph assembly were performed using GraphPad Prism 6 (GraphPad Prism Software). Student's t-test (unpaired, two-tailed) was utilized to compare the means of two groups, while one-way ANOVA (with post hoc analysis) was used to compare the means of three or more groups. ROUT analysis was performed to assess the presence of outsiders. The corresponding figure legends indicate the number of biological samples (n) for each group. Differences in means were considered statistically significant if p < 0.05.

## Results

### Comparison of WI-38 cells with primary lung fibroblasts from idiopathic pulmonary fibrosis patients (LF-IPF)

**Figure 1:**
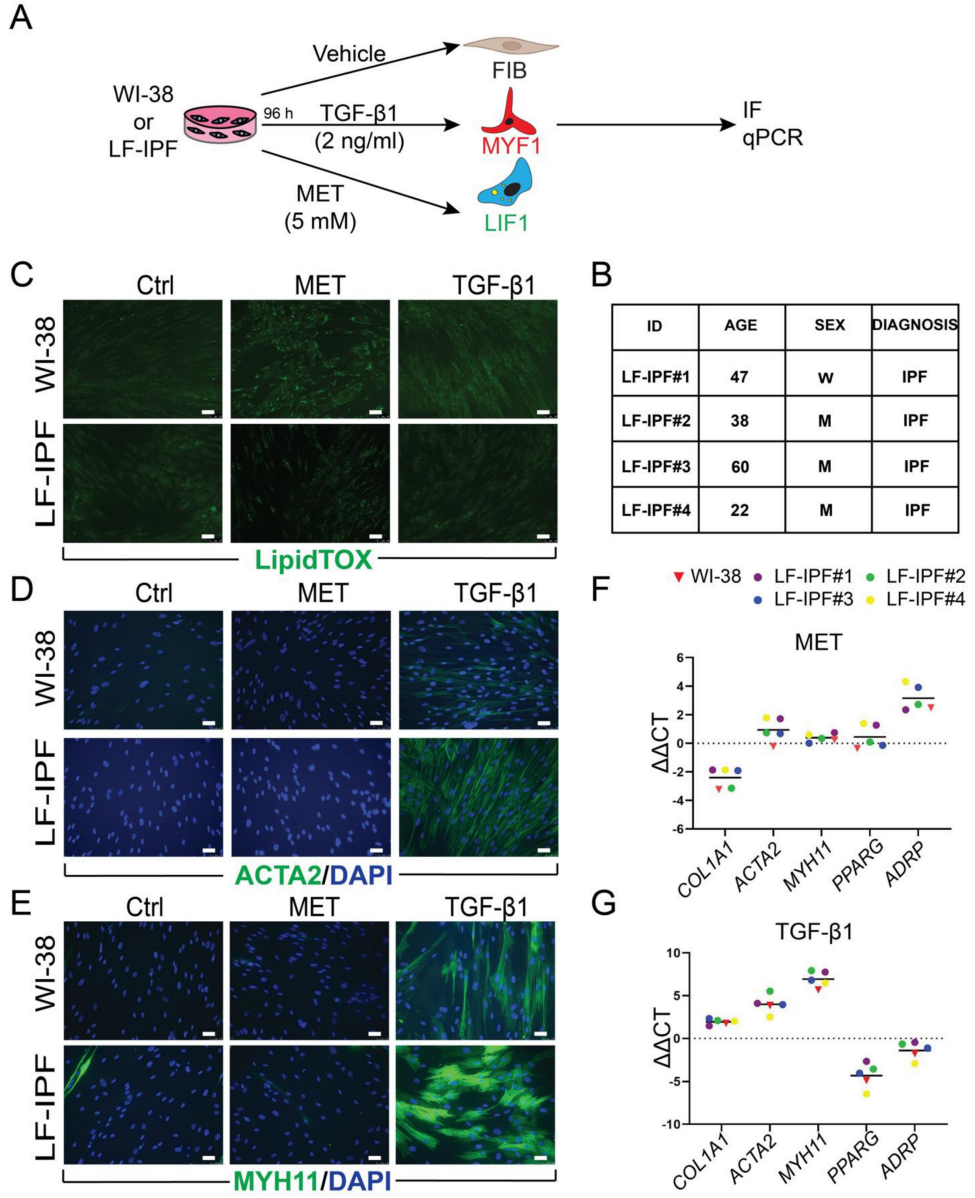
** WI-38 and primary lung fibroblasts from idiopathic pulmonary fibrosis patients (LF-IPF) display similar phenotypical and gene expression response to TGF-β1 and Metformin treatment. (A)** Experimental approach. **(B)** details on the 4 different LF-IPF used for the study. **(C)** IF for LipidTox™. **(D)** IF for ACTA2. **(E)** IF for MYH11. **(F)** qPCR for Metformin vs. vehicle WI-38 and LF-IPF samples. **(G)** qPCR for TGF-β1 vs. vehicle WI-38 and LF-IPF samples. Scale bar C-E: 50 μm.

At first, we focused on the behavior of the WI-38 cells and LF-IPF from four different patients in response to the vehicle, TGF-β1 and Metformin treatment for 96 h, which induce the fibroblast (FIB), myofibroblast (MYF) and lipofibroblast (LIF) phenotypes, respectively (Figure 1A-B). The cells arising from these treatments, called FIB, MYF1 and LIF1, were analyzed by IF and qPCR.

The cells were stained with LipidTox™ to detect the presence of lipid droplets, a hallmark of the LIFs. Upon administration, Metformin-treated WI-38 and LF-IPF cells similarly displayed lipid droplet accumulation within the cellular cytoplasm, characteristic of LIF differentiation, while in the cells treated with TGF-β1, the presence of lipid droplets was limited (Figure 1C). The typical markers of MYFs were detected in TGF-β1-treated cells as elevated ACTA2 and Myosin Heavy Chain 11 (MYH11) expression (Figure 1D-E). WI-38 and LF-IPF cells responded similarly to these treatments.

The quantification of the differentiation efficiency in WI-38 cells was carried out. For LIF differentiation following metformin treatment, we quantified the percentile of DAPI+LipidTox™+ cells over total DAPI+ cells. The induction efficiency for LIF differentiation for the metformin treatment was 95,89%. For the TGF-β1 treatment, we quantified the percentile of DAPI+ACTA2+ cells over total DAPI+ cells. The induction efficiency for the TGF-β1 treatment was 97,71% (data not shown).

Further examination of gene expression by qPCR also highlighted the remarkable similarity between LF-IPFs and WI-38 cells (Figure 1F-G). Metformin-treated WI-38 cells showed a significant increase in *Adipose Differentiation-Related Protein* (*ADRP* Aka* PLIN2*) expression (p = 0.0024 **), consistent with previous research on LF-IPF (Figure 1F) 31. Conversely, TGF-β1 treatment triggered an increase in MYF markers (*ACTA2* p = 0.0027 **, *COL1A1* p = 0.0031 **, *MYH11* p = 0.0078 **) in WI-38 cells, mirroring responses seen in primary fibroblasts (Figure 1G) 32. *Peroxisome proliferator-activated receptor gamma* (*PPARγ*) expression was also reduced upon TGF-β1 treatment, suggesting an attenuation of the LIF phenotype.

### scRNA-seq analysis of LF-IPF treated or not with Metformin

**Figure 2:**
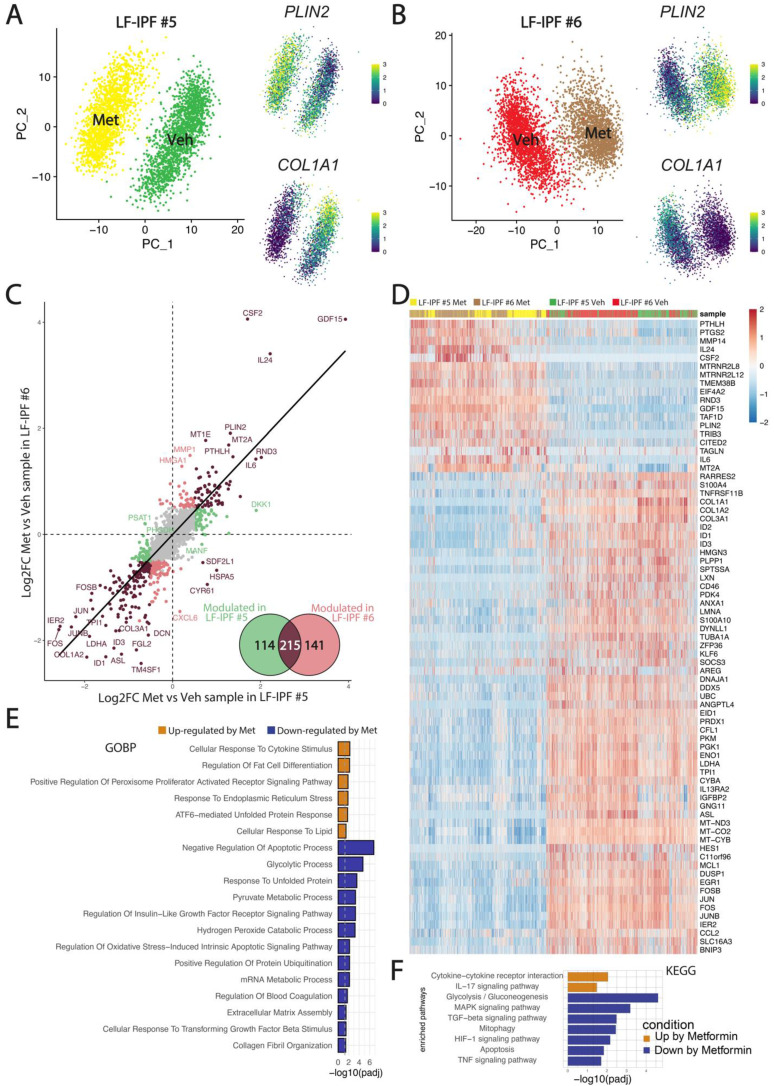
** Impact of Metformin treatment on LF-IPF: a scRNA-seq analysis. (A-B)** UMAP integrating vehicle and Metformin treated LF-IPF for the 2 independent samples considered. Please note that the expression of *COL1A1* and *PLIN2* is drastically decreased and increased by Metformin treatment, respectively. **(C)** Correlation of the Metformin response in the LF-IPF. The log2 Fold change (Metformin/Vehicle) for each gene is plotted. Brown dots correspond to genes significantly modulated in both LF-IPF#5 and LF-IPF#6. Green and red dots correspond to genes significantly modulated in either LF-IPF#5 and LF-IPF#6, respectively. Venn diagram shows the genes modulated by Metformin in the 2 LF-IPF. **(D)** Heatmap integrating the 4 samples showing the main gene markers for each condition. **(E-F)** Functions and pathway enrichment analysis on differentially regulated genes between Metformin and Control. GOBP: Gene Ontology Biological Processes; KEGG: Kyoto Encyclopedia of Genes and Genome pathways.

Next, we evaluated the impact of Metformin treatment on LF-IPF at the single cell level. Two additional independent LF-IPF samples (LF-IPF#5 and LF-IPF#6) were treated with vehicle or 5 mM Metformin in fetal bovine serum-free DMEM. After trypsinization, cells from each sample were labeled with a specific HTO barcoded antibody (CD90), pooled, and simultaneously sequenced using droplet based scRNA-seq (10X Genomics Chromium). Overall, we found a balanced representation for each of the four samples, with more than 2000 cells in each condition and a low percentage of doublets and negative singlets (Figure S1). UMAP integrating vehicle- and Metformin-treated LF-IPF for the 2 independent samples considered are shown in Figure 2A-B indicating that virtually all cells from the 2 distinct samples strongly responded to Metformin. Upon Metformin treatment, the expression of *Collagen Type I Alpha 1 Chain (COL1A1)* is drastically decreased and the expression of *PLIN2* is increased. The Metformin response was highly similar for the two samples indicating that *GDF-15 (Growth Differentiation Factor 15), CSF2 (Colony Stimulating Factor 2)* and *PLIN2* are among the genes positively regulated (Figure 2C-D), with also a strong conservation of a large subset of significantly upregulated genes when compared to WI-38 stimulated cells (Figure S2). Overall, the global signature of Metformin in LF-IPF was strongly associated with a reduction of extracellular matrix organization and a positive regulation of the inflammatory response, cellular response to lipids and endoplasmic reticulum stress (Figure 2E-F).

**Figure 3:**
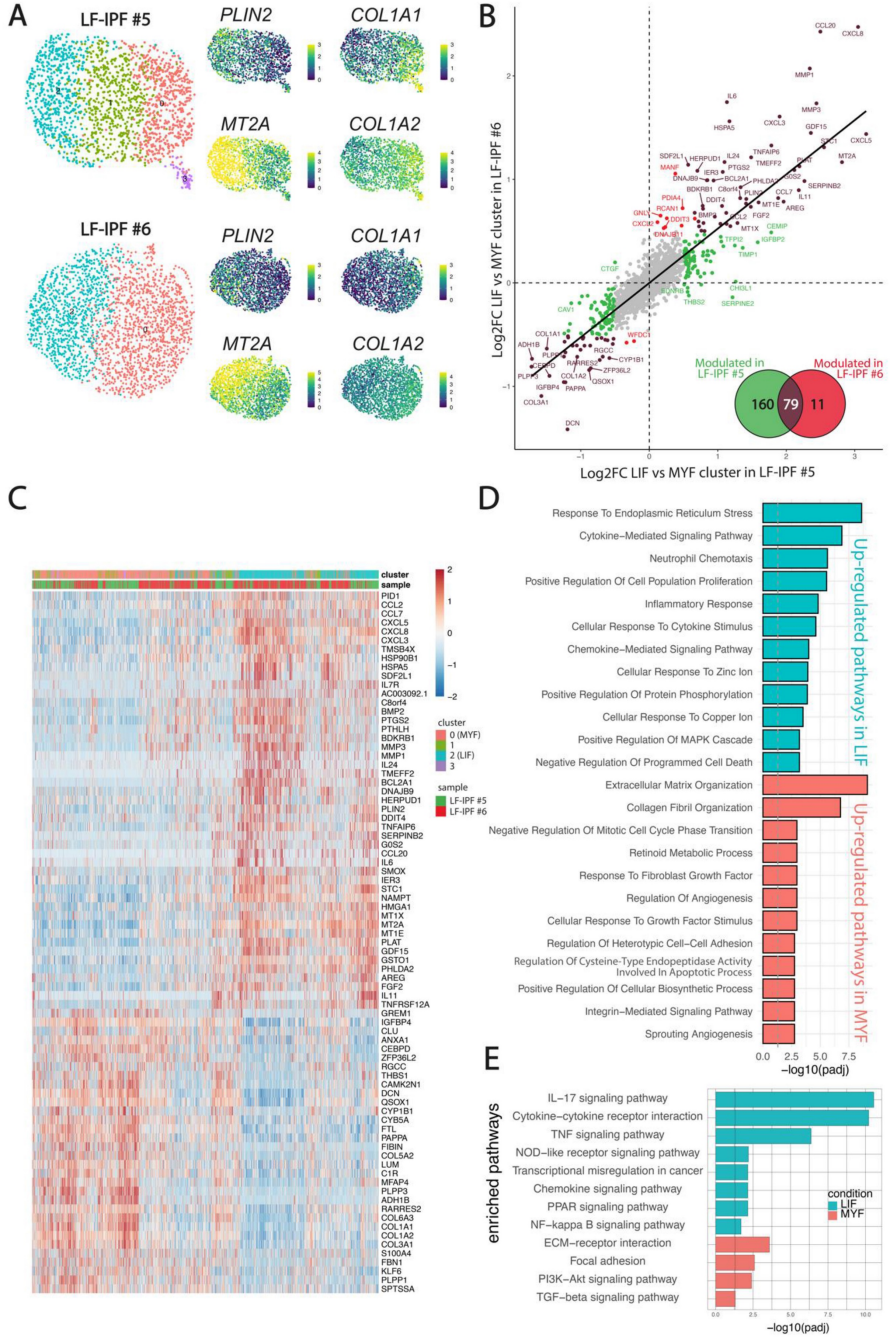
** Evidence for presence of LIF and MYF clusters in non-treated LF-IPF samples. (A)** UMAP for the two independent vehicle-treated LF-IPF. Corresponding heatmap and expression of *COL1A1/COL1A2* and *PLIN2/MT2A* suggest the simultaneous presence of MYF and LIF in LF-IPF samples. **(B)** Correlation of the LIF vs MYF genes in the 2 LF-IPF. The log2 Fold change (MYF vs LIF) for each gene is plotted. Brown dots correspond to genes significantly modulated in both LF-IPF#5 and LF-IPF#6. Green and red dots correspond to genes significantly modulated in either LF-IPF#5 and LF-IPF#6, respectively. The Venn diagram shows the differentially expressed genes between the MYF and the LIF clusters in the 2 LF-IPF. **(C)** Heatmap integrating the 2 samples showing the main gene markers for each subclusters. Note the strong similarity between the LIF and MYF clusters for the 2 independent LF-IPF. **(D-E)** Functions (GOBP) and pathway (KEGG) enrichment analyses on differentially regulated genes between the LIF and the MYF clusters.

Given the previous observation that the LF-IPF samples were heterogenous in terms of MYF and LIF markers, we carried out a fine clustering of the two LF-IPF samples in control conditions. Figure 3A shows the UMAP for the two independent vehicle-treated LF-IPF. Corresponding expression of the *COL1A1* and *PLIN2* on the UMAP suggests the simultaneous presence of MYF and LIF subpopulations in LF-IPF samples. Interestingly, in vehicle-treated LF-IPF#5, we also detected an intermediate cluster (Figure 3A), which is present and named as Fibroblast in the *Haberman dataset* (Figure 5A).

The analysis of the differentially expressed genes in LIF vs MYF subclusters in the two LF-IPF vehicle-treated samples indicated a strong correlation between these subpopulations within the two samples (Figure 3B-C) and revealed, in addition to *PLIN2* the presence of *GDF15* as well as several metallothioneins (*MT1A, MT1E, MT1G, MT1X, MT2A*) in the LIF subpopulation (cluster 2) while genes coding for several collagens (*COL1A1, COL1A2, COL3A1, COL5A2, COL6A3*) were enriched in the MYF population (cluster 0) (Figure 3C). Functional annotation of the 2 clusters confirmed the relevance of these 2 clusters with LIF and MYF, respectively (Figure 3D-E).

Finally, Figure S2 shows a 67 gene signature corresponding to the common genes upregulated by Metformin in both WI-38 cells and LF-IPF and found elevated in the LIF subpopulation (cluster 2) identified in basal LF-IPF.

### Transcriptome profiling and comparative analysis

**Figure 4:**
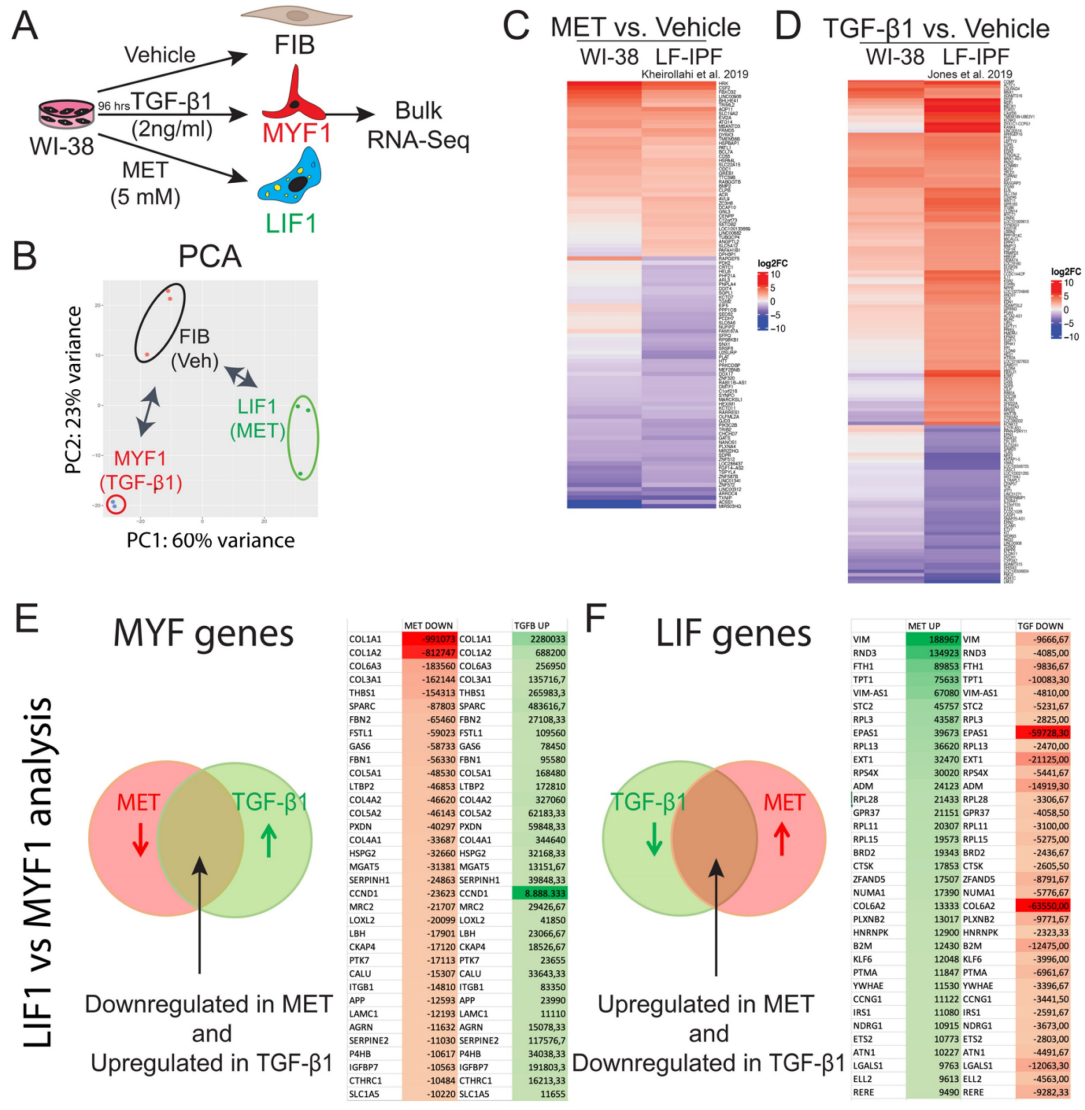
** Bulk RNA-Seq analysis of W-I38 and LF-IPF treated with Metformin or TGF-β1 indicate similar transcriptomic changes. (A)** Experimental approach. **(B)** PCA analysis FIB n = 2, LIF n = 3, MYF n = 2. **(C)** Comparison of Metformin vs Vehicle treatment for WI-38 and LF-IPF using historical data 31. n = 3. **(D)** Comparison of TGF-β1 vs Vehicle treatment for WI-38 and LF-IPF using historical data 32. n = 3. **(E)** Top 35 genes simultaneously upregulated by TGF-β1 and downregulated by Metformin **(F)** Top 35 genes simultaneously downregulated by TGF-β1 and upregulated by Metformin.

To test whether the treated- and non-treated WI-38 cells could be separated by their transcriptional changes, principal component analysis (PCA) was applied to the transcriptome profiles of the total genes obtained by bulk RNA-Seq. PCA revealed clear segregation between these three groups, with Metformin-treated cells exhibiting the most dramatic gene expression changes. Additionally, the first two principal components (PC1 and PC2) can separate the three groups, which explains variations among the samples of 60% and 23%, respectively (Figure 4A-B).

Subsequent comparison of Metformin-treated WI-38 cells with previously published data on Metformin-treated LF-IPF showed significant overlap 31. The derived Heatmap of the top 100 genes revealed the high similarity of the Metformin treated-WI-38 cells with Metformin treated-LF-IPF (Figure 4C, Figure S3). In agreement with the previous result, the comparison between LF-IPF versus WI-38 cells treated with TGF-β1 showed a similar expression pattern in the heatmap (Figure 4D, Figure S3).

We also identified the genes simultaneously upregulated by TGF-β1 and downregulated by Metformin (Figure 4E) and selected the top 35 differentially expressed genes based on the absolute gene expression. We propose that these genes are tightly associated with MYF differentiation. Among them, we found well-recognized fibrosis-associated genes such as *COL1A1* and *COL1A2* as well as *Thrombospondin1* (*THBS1*) 33,34 and *Latent Transforming growth factor β binding protein 2* (*LTBP2*) 35, which are encoding for known regulators of latent TGF-β1 activation and critical players in fibrosis. In addition, we identified *Secreted protein acidic and rich in cysteine* (*SPARC*) 36, encoding a glycoprotein involved in fibrosis, as well as *Follistatin-like 1* (*FSTL1*) 37,38, encoding a glycoprotein sequestering inhibitory ligands of TGF-β, and *Serpin H1* (aka *Hsp47*) 39, encoding a collagen specific molecular chaperone associated with increased collagen accumulation. Less known genes involved in fibrosis found in this list are *Peroxidasin* (*PXDN*) 40, *Growth arrest specific gene 6* (*GAS6*) 41,42, *heparan sulfate proteoglycan* (*HSPG2* aka *Perlecan*) 43, *Alpha-1, 6-Mannosylglycopotein 6-Beta-N-Acetylglucosaminyltransferase* (*MGAT5*) 44 as well as *Lysyl oxidase-like 2* (*LOXL2*) 45,46, encoding an enzyme triggering the network of collagen fibers of the ECM, and *Limb-Bud and Heart* (*LBH*) 47.

Next, we focused on the genes simultaneously downregulated by TGF-β1 and upregulated by Metformin (Figure 4F) and selected the top 35 differentially expressed genes based on the absolute gene expression. We propose that these genes are tightly associated with LIF differentiation. First on this list was *Vimentin* (*VIM*), a gene associated with fibrosis 48. However, Vim-AS1 was also concomitantly expressed, resulting likely in a low level of Vimentin expression in LIFs. Additionally, we found *Rho family GTPase 3* (*RND3*), encoding a primary antagonist of RhoA activity, which promotes fibrosis 49.

Interestingly, Nintedanib and Pirfenidone upregulate *RND3* expression, suggesting that these 2 drugs act via the inhibition of RhoA activity. We also found *Ferritin Heavy Chain 1* (*FTH1*) 50, which plays a role in iron absorption and transportation and participates in the formation of stored iron. When *FTH1* decreases, the production of stored iron decreases, which leads to the accumulation of intracellular Fe2+, inducing ferroptosis, a cell death mediated by iron-dependent lipid peroxidation. Finally, the presence of *Cathepsin K* (*CTSK*) in this list of LIF genes is consistent with the anti-fibrotic nature of the LIFs, as a high level of *CTSK* is associated with decreased collagen deposition and lung resistance following bleomycin treatment 51.

### Comparative analysis with human scRNA-seq samples

**Figure 5:**
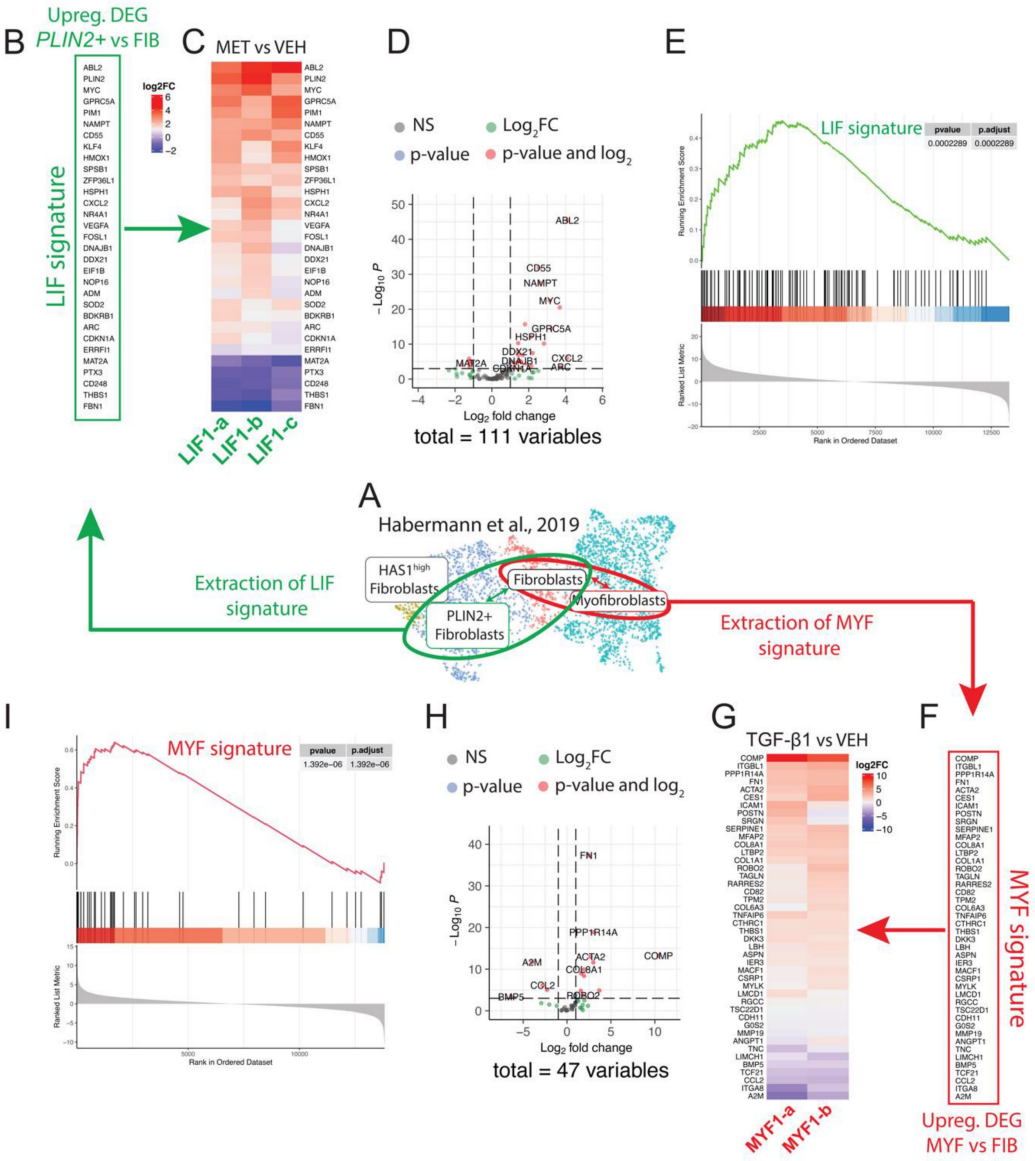
** Conservation of the LIF and MYF signature extracted from Habermann *et al.* scRNA-Seq dataset in WI-38 cells treated with Metformin or TGF-β1, respectively. (A)** UMAP of the Habermann dataset, scRNA-seq showing *PLIN2*+ fibroblasts, Fibroblasts and Myofibroblasts. **(B)** Extraction of the LIF signature by comparing *PLIN2*+ fibroblasts and Fibroblasts. **(C)** Heatmap showing the expression of the LIF signature in Metformin- vs Vehicle-treated WI-38 cells, n = 3. **(D)** Corresponding Volcano plot. **(E)** Corresponding GSEA analysis. **(F)** Extraction of the MYF signature by comparing Myofibroblasts and Fibroblasts. **(G)** Heatmap showing the expression of the MYF signature in TGF-β1 vs Vehicle-treated WI-38 cells, n = 2. H) Corresponding Volcano plot. I) Corresponding GSEA analysis.

We provided supporting data for using the WI-38 cell line as an alternative model to the LF-IPFs to study LIF and MYF differentiation. To better characterize our model, we extrapolated the LIF and MYF signature obtained from the Habermann scRNA-seq dataset previously published, where 5 Donors and 20 IPF lungs were analyzed (Figure 5A). The expression of the top expressed genes between *PLIN2*+ fibroblasts and Fibroblasts allows to identify 31 genes enriched in *PLIN2+* fibroblasts, including *PLIN2*, *C-X-C Motif Chemokine Ligand 2* (*CXCL2*), *MYC Proto-Oncogene (MYC)* and *G Protein-Coupled Receptor Class C Group 5 Member A* (*GPRC5A*) (Figure 5B). The expression of these selected genes representing the LIF signature was then investigated in the Metformin vs. Vehicle bulk RNA-seq data (n = 3 independent comparison) from WI-38 cells (Figure 5C). Our results indicated a remarkable upregulation of the LIF signature upon Metformin treatment. The Volcano plot shows the upregulated and downregulated genes in WI-38 cells treated with Metformin using an extended LIF signature containing the top 111 genes expressed (Figure 5D). The GSEA plot for the LIFs indicates a high correlation (p = 0.0002289) in the enrichment of the genes identified in the Habermann dataset (*PLIN2*+ fibroblasts vs Fibroblasts) and WI-38 cells treated with metformin (Figure 5E).

In Figure 4 and 5, we identified and prioritize based on their level of expression, MYF genes (genes simultaneously upregulated with TGF-β1-treated WI-38 cells and in Myofibroblasts from the Habermann dataset) as well as LIF genes (genes simultaneously upregulated with Metformin-treated WI-38 cells and in *PLIN2+* fibroblasts from Habermann dataset). Within these two lists, we identified the transcription factors potentially involved in differentiation (either positively or negatively) and/or maintenance of the MYF or LIF.

Among the transcription factors playing a potential role in the differentiation and/or maintenance of the LIF, we found *Krüppel-like factor 4* (*KLF4*) and *Nuclear Receptor Subfamily 4 Group A Member 1 (NR4A1)*. KLF4 is a transcription factor highly increased in *PLIN2*+ fibroblasts compared to fibroblasts (Habermann dataset). Deletion of *Klf4* in ACTA2+ myofibroblasts in adult mice before bleomycin administration leads to increased levels of collagens, α-SMA, COL1, FN1 and proliferation and accumulation of myofibroblast 14 days after bleomycin administration 52. Ubiquitous overexpression in transgenic mice of *Klf4* inhibited bleomycin-induced fibrosis formation and the accumulation of collagen fibers 53. Furthermore, overexpression of *KLF4* in MRC-5 fibroblasts pre-treated with TGF-β1 reverses their myofibroblasts phenotype (decreased expression of α-SMA and COL1A2) 54. These studies therefore support a key role for KLF4 in the negative control of MYF differentiation and maintenance. Based on the fact that KLF4 was identified as a LIF gene, we propose that KLF4 is instrumental in MYF to LIF differentiation (which will be the topic of future studies using WI-38 cells as in Figure 7).

Our preliminary results validate our silencing strategy for *KLF4* (data not shown). A trend in the decrease of *ADRP* is observed upon silencing of *KLF4* in vehicle-treated WI-38 cells. Further studies in the context of metformin treatment will be needed to confirm the role of KLF4 in the maintenance of the LIF phenotype.

We also found that the transcription factor NR4A1 was increased in *PLIN2*+ fibroblasts. A model of skin fibrosis induced by overexpressing a constitutively active form of the TGF-β receptor I was carried out. Activation of TGF-β1 signaling in the context of *Nr4a1^-/-^* mice led to a massive deposition of collagen and increased presence of myofibroblasts. In the context of a lung fibrosis model induced by bleomycin, *Nr4a1^-/-^* mice displayed increased expression of *Col1a1*, *Col1a2* together with increased hydroxyproline content in the lung 55.

Therefore, both KLF4 and NR4A1 identified in our list as LIF genes are good candidates to prevent MYF differentiation (negative regulators). Therefore, KLF4 and NR4A1 may be important for the FIB to LIF differentiation (as in Figure 1).

The pathways involved in LIFs were related to the immune system, degradation of the extracellular matrix, collagen biosynthesis and modifying enzymes and others (Figure S4A).

On another side, the expression of the top expressed genes between MYFs and FIBs allows us to identify 47 genes enriched in MYFs, including *Cartilage Oligomeric Matrix Protein* (*COMP), Carboxylesterase 1 (CES1), ACTA2, Integrin Subunit Beta Like 1 (ITGBL1), COL1A1*, and *Collagen Triple Helix Repeat Containing 1 (CTHRC1)* (Figure 5F). The expression of these selected genes representing the MYF signature was then investigated in the TGF-β1 vs. Vehicle bulk RNA-Seq data (n = 2 independent comparison) from WI-38 cells (Figure 5G). We observed an evident upregulation of the MYF signature in WI-38 cells upon TGF-β1 treatment. The Volcano plot indicates the upregulated and downregulated genes in WI-38 cells treated with TGF-β1 using an extended MYF signature containing the top 47 genes expressed (Figure 5H). Similar to the GSEA plot in LIF, the GSEA plot in MYF indicates a significant high correlation (p = 1,392 10^-6^) in the enrichment of the genes identified in the Habermann dataset (Myofibroblasts vs Fibroblasts) and WI-38 cells treated with TGF-β1 (Figure 5I).

Among the transcription factors playing a potential role in the differentiation and/or maintenance of the MYF, we found *LBH*, a transcription factor involved in the WNT signaling 56. Silencing of *LBH* in hepatic stellate cells, before the TGF-β1 stimulus, decreases the expression of markers linked to hepatic stellate cells activation during liver fibrosis such as α-SMA and COL1A1 57. In cardiac fibroblasts, overexpression of *LBH* promotes fibroblast to myofibroblast transition supported by increased expression of α-SMA and COLLAGEN 1, together with organized stress fibers structures 47.

Our preliminary results (data not shown) validate the silencing of *LBH* using our siRNA approach. Silencing of *LBH* in vehicle-treated WI-38 cells tends to decrease *ACTA2* expression. Furthermore, silencing of *LBH* during TGF-β1 treatment leads to the decrease in *ACTA2* and *MYH11* expression*.* These results therefore suggest that LBH is critical for MYF maintenance.

In agreement to the known role of myofibroblasts in fibrosis, the pathways enriched in MYFs were related to the integrin cell surface interactions, ECM proteoglycans, collagen chain trimerization, extracellular matrix organization and others pathways (Figure S4B).

### Functional assays and phenotypic changes

**Figure 6:**
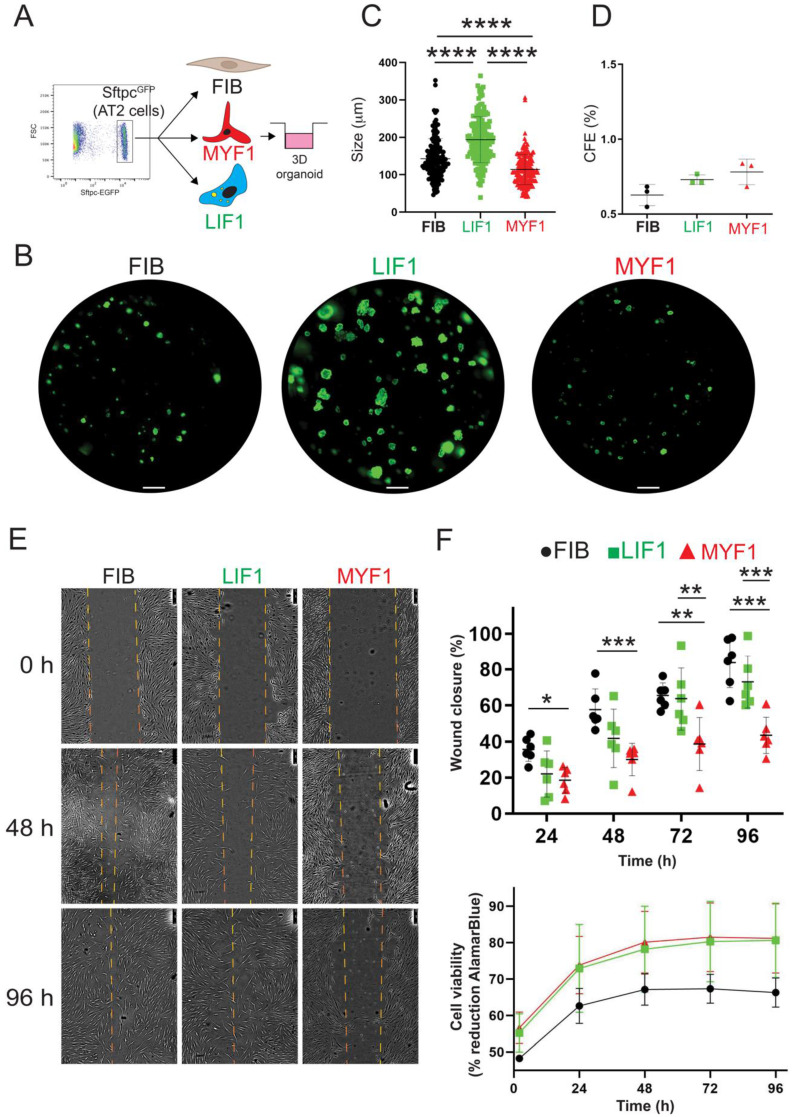
** Alveolosphere assays indicate that LIF1 enhance organoid growth while MYF1 elicit organoid growth inhibition. (A)** Experimental approach. **(B)** Organoids generated with FIB, LIF1 and MYF1. Quantification of organoid **(C)** size and **(D)** Colony forming efficiency, n = 3. **(E)** Scratch assay. **(F)** Wound closure quantification and cell viability n = 6. *P* values * *P* < 0.05; ** *P* < 0.01; *** *P* < 0.001; **** *P* < 0.0001. Scale bar B: 1000 μm, E: 250 μm.

To functionally characterize the capacity of the Metformin- and TGF-β1-treated WI-38 cells to sustain AT2 proliferation, we performed an alveolosphere assay. This assay was carried out by mixing in Matrigel, FIB, LIF1 or MYF1 cells with mouse AT2 cells isolated by FACS using Sftpc^GFP^ lungs (Figure 6A, Figure S5A). Alveolosphere formation is observed within 14 days, as shown in Figure 6B. Quantification of the size of the organoids indicated that LIF1 (on average 193.9 µm) are significantly supporting the growth of AT2 cells compared to FIB (on average 142.8 µm) or MYF1 (on average 114 µm) (Figure 6C). No significant difference was observed in the colony forming efficiency between these 3 populations of WI-38 cells (Figure 6D). Taken together, these data are emphasizing the supporting role of LIF1 on AT2 survival and proliferation, illustrated by the increase in the spheroid size in LIF1 vs. MYF1.

Additionally, the Metformin-treated cells (LIF1) demonstrated an enhanced migratory capacity in scratch tests compared to TGF-β1-treated cells (MYF1) at 72 h and 96 h. MYF1 migration capabilities were severely compromised, reflecting a phenotypical feature of fibrotic tissue (Figure 6E-F). These results underscore the practical implications of the LIF and MYF phenotype induced in WI-38 cells. Finally, Alamar Blue viability assay on WI-38 cells treated with Metformin and TGF-β1 revealed no differences between the two groups (Figure 6F), indicating that the treatments did not impact cell viability.

### Evidence for MYF to LIF differentiation

**Figure 7:**
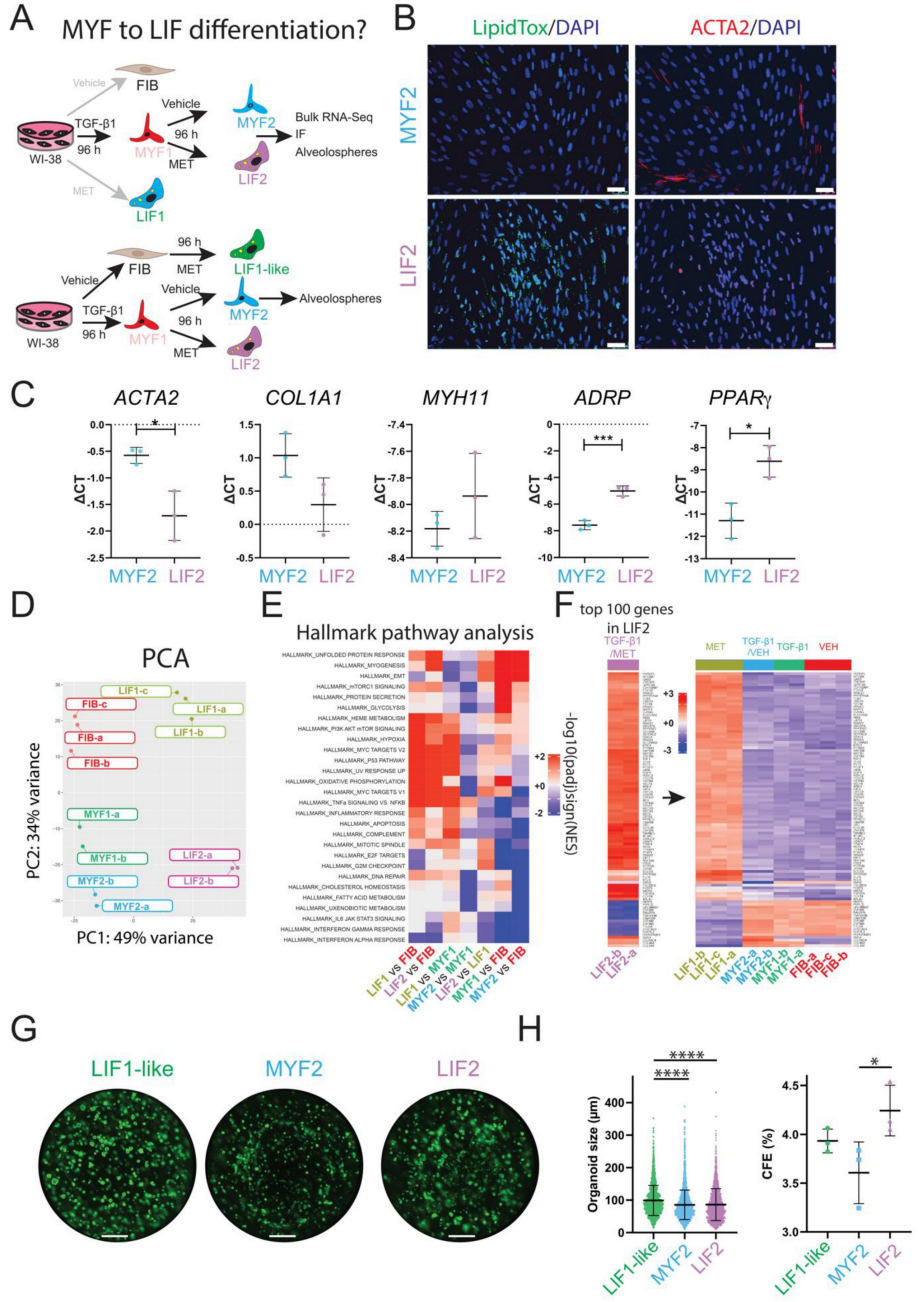
** Evidence for MYF to LIF differentiation**. **(A)** Experimental approach. **(B)** IF for LipidTox™ and ACTA2 in MYF2 and LIF2, n = 3. **(C)** qPCR expression of MYF and LIF markers, n = 3. **(D)** PCA graph comparing FIB, LIF1, MYF1, LIF2 and MYF2. **(E)** Hallmark pathway analysis. **(F)** Heatmap showing the expression of the top 100 regulated genes in LIF2 and the expression of these genes in LIF1, MYF2, MYF1 and FIB. **(G)** An alveolosphere assay shows the organoids corresponding to co-culture of LIF1-like, LIF2, and MYF2 with Sftpc^GFP+^ cells at day 14. **(H)** Quantification of organoid size and colony formation efficiency, n = 3. *P* values * *P* < 0.05; ** *P* < 0.01; *** *P* < 0.001; **** *P* < 0.0001. Scale bar B: 50 μm and G:1250 μm.

Our results confirm the effectiveness of sequential TGF-β1 and Metformin treatment in inducing the MYF to LIF transition in WI-38 cells. After introducing Metformin or vehicle following TGF-β1 treatment, the cells generated after 8 days in culture are called LIF2 and MYF2 (Metformin- and vehicle-treated, respectively) (Figure 7A). We monitored the expression of MYF and LIF markers in the newly differentiated LIF2 vs MYF2 by IF and qPCR. Our results show a decrease in ACTA2 and an increase in lipid droplet accumulation (Figure 7B). Notably, the expression of *ACTA2* was reduced while the expression of *ADRP* and *PPARγ* was increased, correlating with the MYF to LIF transition following Metformin treatment in our cell line (Figure 7C).

To better define the nature of these new LIF2 and MYF2 fibroblasts, we investigated the global transcriptomic changes occurring in these cells by bulk RNA-Seq. The resulting PCA graph compares LIF2 and MYF2 with the previously generated transcriptomic data for FIB, LIF1 and MYF1 (Figure 7D). Our data indicate that LIF2 are different from LIF1, suggesting that the MYF to LIF differentiation is incomplete. Interestingly, MYF2 appear to display a more significant difference with FIB than MYF1, suggesting a potentially enhanced MYF phenotype.

We also carried out hallmark pathway analysis to compare the different populations to each other (Figure 7E). The comparison of LIF1 vs FIB indicated increased Heme Metabolism, PI3K AKT mTOR signaling, Hypoxia, MYC targets, TNFα signaling vs NFκB as well as decreased Interferon alpha response. Similar regulations for these pathways were observed in LIF2 vs FIB. Direct comparison of LIF2 vs LIF1 indicates decreased TNFα signaling vs NFκB, inflammatory response, apoptosis, complement, IL6/JAK/STAT3, interferon Gamma and Alpha responses. These pathways are also decreased in MYF1 vs FIB and MYF2 vs FIB suggesting that LIF2 represent an intermediate between MYF1 and LIF1. LIF2 are not fully differentiated into a LIF1, still displaying some MYF characteristics.

Additionally, we identified the top 100 genes regulated in LIF2 and examined the expression of these genes in LIF1, MYF2, MYF1 and FIB (Figure 7F, Figure S6 for high magnification of the heatmap). Our results indicated a conservation of these top-regulated genes in LIF1, supporting the MYF to LIF reversion. Moreover, these genes showed opposite regulation in MYF2, MYF1 and FIB, indicating that LIF2 display a different differentiation status compared to the other phenotypical variants.

Finally, we assessed the capacity of LIF2 and MYF2 to support alveolosphere formation as previously described. For this assay and in order to match exactly the same *in vitro* culture time as MYF2 and LIF2, we included LIF1-like cells (96 h vehicle-treated fibroblasts, then 96 h metformin-treated fibroblasts) as control (Figure 7G). LIF1-like cells displayed the highest organoid size (on average 99.13 µm) compared to LIF2 (on average 86.28 µm) and MYF2 (on average 85.52 µm). However, the organoid size generated with LIF2 and MYF2 was not different (Figure 7H). Interestingly, LIF2 displayed a significant increase in the CFE compared to MYF2, suggesting that LIF2 confers a survival advantage to the AT2 cells but does not impact their proliferation. Altogether, the difference between LIF2 and MYF2 CFE, indicated that LIF2 has partially reversed to a phenotype supportive of alveolosphere formation.

### Evidence for LIF to MYF differentiation

**Figure 8:**
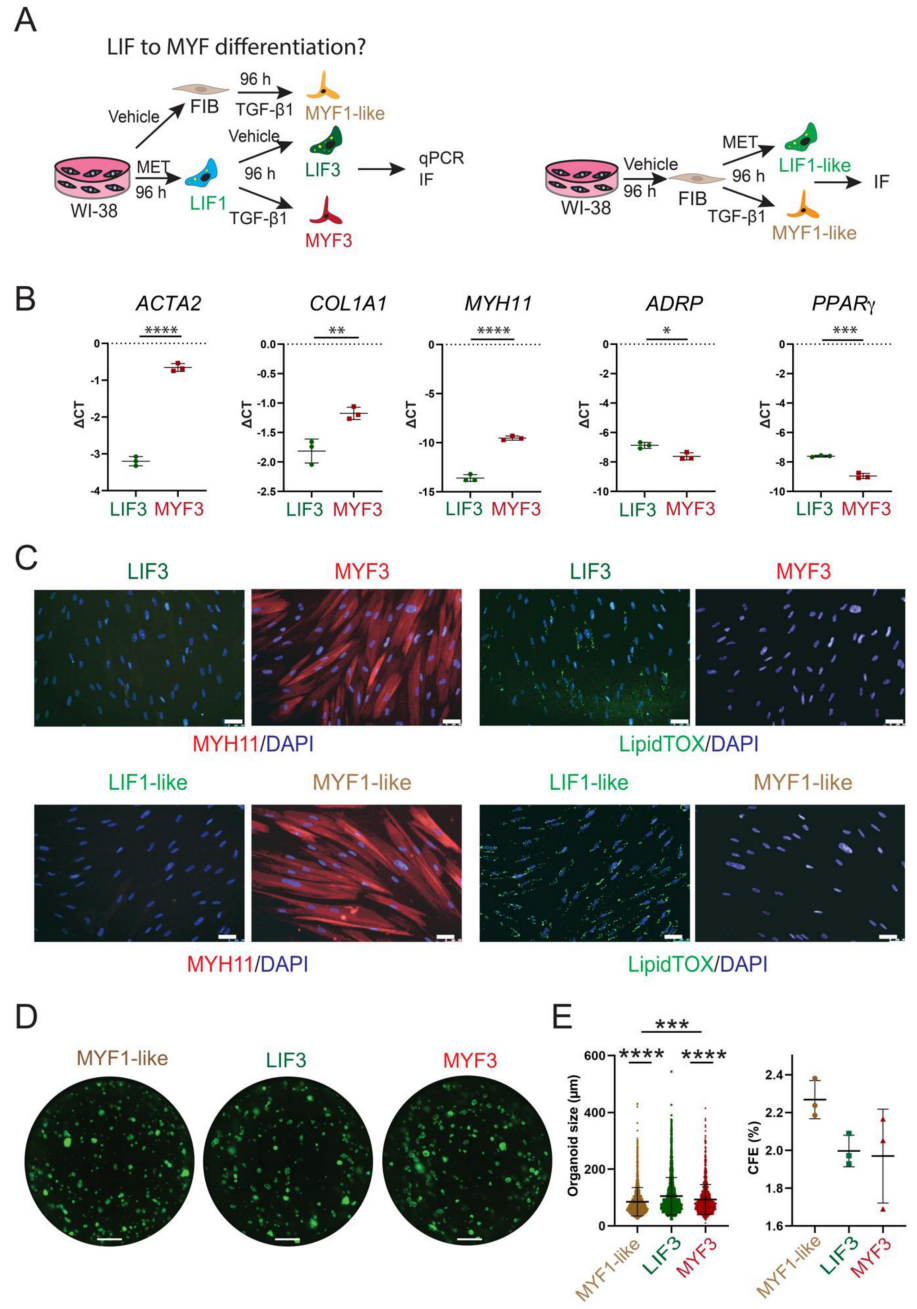
** Evidence for LIF to MYF differentiation. (A)** Experimental approach. **(B)** qPCR expression of MYF and LIF markers, n = 3. **(C)** IF for MYH11 and LipidTox™, n = 3. **(D)** An alveolosphere assay shows the organoids corresponding to co-culture of MYF1-like, LIF3, and MYF3 with Sftpc^GFP+^ cells at day 14. **(E)** Quantification of organoid size and colony formation efficiency, n = 3. *P* values * *P* < 0.05; ** *P* < 0.01; *** *P* < 0.001; **** *P* < 0.0001. Scale bar C: 50 μm and D: 1250 μm.

We previously reported that LIF served as a source of activated MYFs during fibrosis formation 58. To evaluate this, we investigated the LIF to MYF differentiation by pre-treating the WI-38 cells with Metformin followed by TGF-β1 or Vehicle. The cells generated after 8 days in culture are called MYF3 and LIF3 (Figure 8A). First, we monitored the expression of MYF and LIF markers in LIF3 vs MYF3 by qPCR and IF. *ACTA2*, *COL1A1,* and *MYH11* expressions were significantly increased, while the expressions of *ADRP* and *PPARγ* were significantly decreased in MYF3 (Figure 8B).

An increase in MYH11 and decreased in LipidTox™ signal related to the lipid droplets were observed in MYF3. In contrast to MYF3, scarce signal of MYH11 and presence of LipidTox™ signal was observed in LIF3 (Figure 8C). LIF1-like and MYF1-like (96 h vehicle-treated cells followed by either 96 h Metformin- or TGF-β1-treatment) were used as controls.

Next, we assessed the capacity of LIF3, MYF3 and MYF1-like cells to support alveolosphere formation (Figure 8D). As expected, MYF1-like cells displayed the lowest organoid size (on average 85.26 µm) compared to LIF3 (on average 105.2 µm) and MYF3 (on average 93.5 µm). The CFE was not significantly different between these conditions (Figure 8E). Altogether, these results support the LIF to MYF transition in WI-38 cells.

## Discussion

The heterogeneity of fibroblast subpopulations and their dysregulation in conditions such as IPF contribute to tissue structure loss and alveolar reduction. Specifically, myofibroblast activation is crucial in collagen deposition and extracellular matrix remodeling. The MYF population is one of the most extensively studied in the context of fibrosis. However, technical limitations associated with *in vitro* models, such as primary fibroblast cultures, hinder a complete understanding of these cells.

To overcome the limitations associated with primary fibroblast cultures from patients, our study validates the WI-38 cell line, a human embryonic diploid fibroblast cell line, as an *in vitro* model for investigating MYF differentiation upon TGF-β1 treatment. Additionally, we utilized Metformin, an anti-diabetic drug, to induce LIF differentiation in WI-38 cells, in accordance with a previous study 31. Previous research has shown the ability of these cells to differentiate into LIF and MYF after treatment with parathyroid hormone-related protein (PTHrP) 18,59 and TGF-β1, respectively 19.

WI-38 cells display tremendous technical advantages, specifically in terms of maintenance and culture conditions, genetic manipulation capabilities, and data availability for comparative analysis. One of the most significant advantages of the WI-38 cell line over LF-IPFs pertains to their maintenance and culture conditions. While primary cells often necessitate stringent conditions, such as specialized growth media and carefully controlled environmental factors, WI-38 cells are relatively easy to maintain and culture. The WI-38 cells have a higher propagation capacity, undergoing up to 50 duplications, significantly more than primary fibroblasts. This makes them ideal for long-term experiments, where a continuous supply of cells is essential, thereby minimizing the need for fresh primary cultures and providing a viable solution for their scarcity as well as for the ethical issues associated with their use.

Another crucial advantage of the WI-38 cell line is its amenability to genetic manipulation, a feature that is often more challenging in primary cultures. The existence of specialized kits designed for the transfection of WI-38 cells further facilitates the process. This not only streamlines the workflow but also opens up new avenues for research, allowing for more complex experimental designs. Such genetic manipulation capabilities are less straightforward in primary cells, which often require more cumbersome techniques and have lower transfection efficiencies. The increasing use and validation of WI-38 cells in multiple studies have led to a wealth of data that can be leveraged for comparative analyses. The consistent genetic background of this cell line makes it easier to interpret and compare data across different studies and laboratories. This is in stark contrast to LF-IPFs, where the genetic variability inherent in primary isolates often leads to batch-to-batch differences, complicating data interpretation and hindering the reproducibility of results. The availability of a standardized, well-characterized cell line like WI-38 enhances the robustness and reliability of data, thus fortifying its utility in large-scale, multi-center studies.

Beyond using WI-38 cells to study MYF differentiation, we report that Metformin-treated WI-38 cells exhibit characteristic features of LIF differentiation, including the accumulation of lipid droplets. LIFs, initially discovered during lung development, were previously regarded as supporting cells for AT2 and a source of lipids for surfactant protein (SFTPC) production. However, recent studies have revealed their active role in supporting AT2 cell proliferation. In the case of lung fibrosis, TGF-β1 induces MYF differentiation in fibroblasts, and LIFs can transdifferentiate into MYFs. However, MYFs cannot support AT2 cell proliferation and differentiation, resulting in alveolarization loss.

In our work, we demonstrated that Metformin treatment triggered the accumulation of lipid droplets in the cytoplasm of both LF-IPFs and WI-38 cells, indicating LIF differentiation. The expression of *ADRP* significantly increased upon Metformin treatment, supporting Metformin's role in lipid accumulation and LIF differentiation.

Concurrently, TGF-β1 treatment amplified MYF-specific markers ACTA2 and MYH11 expression in both LF-IPFs and WI-38 cells, indicating successful induction of the MYF phenotype. Immunofluorescence and gene expression analysis confirmed the differentiation of WI-38 cells into MYFs and LIFs upon TGF-β1 and Metformin treatment, respectively. Notably, the expression levels of *ACTA2*, *COL1A1*, and *MYH11*, considered markers for MYFs and activated myofibroblasts, were comparable between WI-38 cells and LF-IPFs. LIF differentiation after Metformin treatment also confirmed the similarity between WI-38 cells and LF-IPFs, with the accumulation of lipid droplets and the gene expression of *ADRP* and *PPAR*γ overlapping in both models.

Interestingly, it was previously reported that PPARγ was activated upon TGF-β1 challenge in alveolar macrophages 60. We propose that the reduction in PPARγ expression in WI-38 cells in response to TGF-β1 is linked to the different nature of the cells (fibroblast versus macrophages).

Furthermore, sequential treatment of WI-38 cells first with TGF-β1 and then with Metformin led to MYF to LIF transdifferentiation, (Figure 7) underscoring the pivotal role of Metformin in this cellular reprogramming (for a summary of all the experimental conditions see Figure S5B). We are also providing evidence for LIF to MYF differentiation (Figure 8) supporting the LIF-MYF reversible switch model 58.

Comparison between scRNA-seq data from human samples and our *in vitro* model further demonstrated the effectiveness of this approach. The gene expression signatures observed in LIF and MYF cells in our *in vitro* WI-38 model showed a remarkable overlap with human lung samples. This further validates our model and, importantly, the therapeutic potential of Metformin.

Interestingly, our transcriptome analysis revealed a profound impact of Metformin treatment on the gene expression of WI-38 cells. The significant enrichments in the KEGG pathways highlighted Metformin's effects on metabolic reprogramming and lipid trafficking. However, the exact molecular mechanism by which Metformin achieves this remains a subject for future investigation.

In the organoid formation assay, Metformin-treated WI-38 cells supported alveolosphere formation more effectively, indicating that the LIF phenotype induced by Metformin could significantly impact lung tissue repair. However, fibroblast viability was comparable among all the groups, demonstrating that these therapeutic alterations do not compromise the fundamental properties of the cells.

In conclusion, our results provide compelling evidence of Metformin's role in lung fibroblast differentiation and its therapeutic potential. The high comparability between the WI-38 cell line and LF-IPFs will allow further study of LIF and MYF differentiation and explore potential therapies for conditions such as pulmonary fibrosis. In addition, our findings emphasize that while Metformin's role in the lung is still not fully understood, it offers an exciting possibility as a therapeutic tool. Furthermore, the WI-38 cell line offers compelling advantages over primary fibroblasts, making it a valuable resource for studies focused on lung homeostasis and disease. Its ease of maintenance, higher propagation capacity, and capability for genetic manipulation provide researchers with a versatile and reliable model system. Additionally, the wealth of comparative data available for WI-38 cells enhances its utility, making it a preferred choice for basic and applied research in lung fibroblast differentiation and fibrotic lung diseases. The substantial evidence gathered through the WI-38 cell model provides a robust foundation for further exploration of Metformin's potential roles in lung repair and fibroblast differentiation.

Therefore, our study strongly advocates for the broader adoption of the WI-38 cell line in future research endeavors.

## Supplementary Material

Supplementary figures.

## Figures and Tables

**Table 1:** Primer sequences used for qPCR analysis.

Gene	Forward primer sequence	Reverse primer sequence
ACTA2	5' -CTG TTG CAG CCA TCC TTC AT- 3'	5' -TCA TGA TGC TGT TGT AGG TGG- 3'
ADRP	5' -TCA GCT CCA TTC TAC TGT TCA CC- 3'	5' -CCT GAA TTT TCT GAT TGG CAC- 3'
COL1A1	5' -ATG TTC AGC TTT GTGGAC CTC- 3'	5' -CTG TAC GCA GGT GAT TGG TG- 3'
PPARγ	5' -TTG CTG TCA TTA TTC TCA GTG GA- 3'	5' -GAG GAC TCA GGG TGG TTC AG- 3'
MYH11	5' -GCA GCT ACA GGC TGA AAG GA- 3'	5' -CCT CCA GCT GTT CTT CAA GG- 3'
GAPDH	5' -GAA AGC CTG CCG GTG ACT AA- 3'	5' -GCC CAA TAC GAC CAA ATC AGA G- 3'

**Table 1:** List of the forward and reverse primers used in the qPCR analysis: *ACTA2*, *COL1A*, and *MYH11* as MYF markers; *ADPR*, and *PPARγ* as LIF markers. *GAPDH* serves as a housekeeping gene.

**Table 2:** Nucleotide sequence of the 4 HTOs used for Cell Hashing.

HTO_BC1: AGGACCATCCAA
HTO_BC2: ACATGTTACCGT
HTO_BC3: AGCTTACTATCC
HTO_BC4: TCGATAATGCGA
