# Supplementary material for: Highlighting fibroblast plasticity in lung fibrosis: the WI-38 cell line as a model for investigating the myofibroblast and lipofibroblast switch
Source: Theranostics. 2024 Jun 11;14(9):3603–22. doi: 10.7150/thno.93519 (PMC11209726; doi:10.7150/thno.93519)
Supplement: Supplementary file 1 — Supplementary figures. [file thnov14p3603s1.pdf]

Highlighting fibroblast plasticity in lung fibrosis: the WI-38 cell line as a model for investigating the myofibroblast and lipofibroblast switch

Esmeralda Vásquez-Pacheco[1*], Manuela Marega[1,2*], Arun Lingampally[1], Julien Fassy[3], Marin Truchi[3], Kerstin Goth[1], Lisa Trygub[1], Sara Taghizadeh[1,4], Marek Bartkuhn[1,4], Ioannis Alexopoulos [1,4], Ying Dong[1], Kevin Lebrigand[3], Andreas Gunther[1,4], Chengshui Chen[5,6], JinSan Zhang[5,6], Cho-Ming Chao[1,7], Denise Al Alam[8], Elie El Agha[1,4], Bernard Mari[3], Saverio Bellusci[5,9,#], Stefano Rivetti[1,#]

Supplementary figures

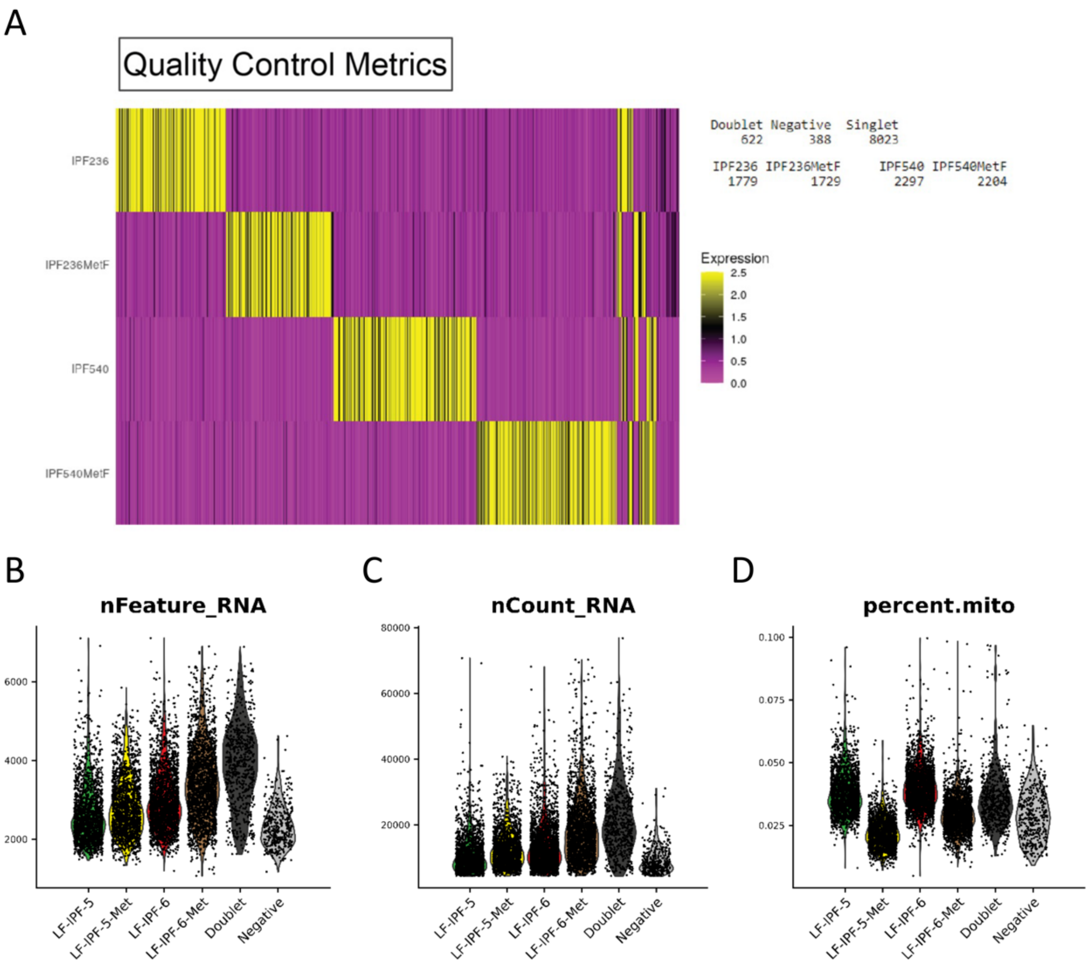

Figure S1: Quality control metrics of the scRNA-seq experiment. A) Heatmap of HTO counts in each cell, labelled according to assigned experimental condition after demultiplexing. Violin plots showing **B**) the number of genes detected in each cell, **C**) the number of UMI per cell and **D**) the percentage of cell reads originating from the mitochondrial genes.

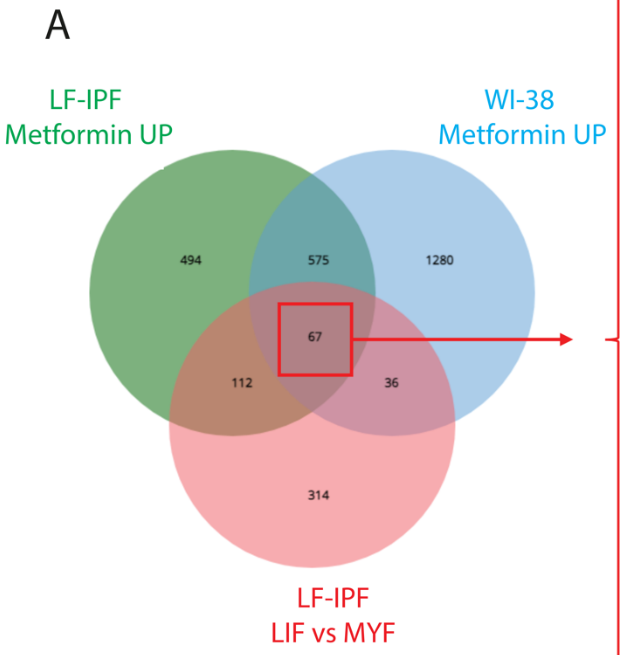

B

genes	WI-38 Met UP	LF-IPF Met UP	LF-IPF LIF vs MYF
AGO2	2.95	0.19	0.11
AHR	1.69	0.54	0.17
ARF1	0.91	0.41	0.20
ATP2B1	0.62	0.83	0.31
BCL2A1	5.02	0.87	0.82
BIRC3	3.11	0.37	0.19
BRIX1	1.46	0.32	0.15
BTG3	2.24	0.95	0.23
CCT8	1.07	0.20	0.24
CEBPB	3.42	0.62	0.33
COQ10B	1.26	0.18	0.12
COX17	1.45	0.38	0.15
CXCL8	2.90	0.81	2.81
DDIT3	2.90	1.54	0.33
DDX21	1.38	0.48	0.36
DIEXF	2.50	0.24	0.15
DNAJB9	2.12	1.00	0.77
EIF1B	1.44	0.47	0.18
ESF1	1.79	0.51	0.14
ETS2	1.49	0.35	0.14
FAM60A	1.41	0.10	0.11
G0S2	2.39	1.24	1.46
GDF15	5.73	3.93	1.60
GEM	3.12	1.07	0.23
IER3	1.79	0.72	0.83
IFRD2	1.52	0.12	0.17
IMP4	1.37	0.29	0.16
MED30	1.66	0.11	0.18
MLX	1.05	0.20	0.11
MYC	3.12	0.35	0.40
NAMPT	2.54	1.13	0.84
NINJ1	1.04	0.22	0.24
NR3C1	1.28	0.14	0.20
NTMT1	1.57	0.47	0.16
NXT1	2.14	0.18	0.18
ODC1	2.23	0.17	0.13
OSGIN2	3.59	0.33	0.25
PA2G4	1.04	0.26	0.21
PAK1IP1	1.73	0.18	0.13
PFDN2	1.54	0.42	0.28
PIM3	4.08	0.80	0.21
PLIN2	3.68	1.31	0.80
PNO1	1.54	0.16	0.16
PTGS2	4.52	1.31	1.01
RAB32	1.26	0.65	0.32
RABGGTB	1.74	0.79	0.26
RASSF8	1.54	0.22	0.21
RELB	1.97	0.32	0.12
RIPK2	1.59	0.63	0.34
RNF126	1.96	0.18	0.13
RNPS1	0.75	0.21	0.11
RPF2	1.92	0.51	0.18
RPL22L1	1.55	0.53	0.32
RSL1D1	1.79	0.33	0.16
SDCBP	1.94	0.94	0.28
SLC16A7	1.47	0.18	0.19
SLC3A2	3.88	0.84	0.24
SMS	0.76	0.56	0.35
SNHG15	2.54	0.17	0.27
SRP72	0.66	0.14	0.13
STC2	1.60	0.61	0.18
TEX30	0.95	0.34	0.14
UBA2	0.92	0.12	0.17
UPP1	2.62	0.34	0.34
USP53	2.12	0.33	0.12
UTP18	1.16	0.11	0.13
WDR43	1.84	0.15	0.14

Figure S2: Identification of a cluster of 67 genes commonly upregulated in LIF vs MYF subclusters in LF-IPF (scRNAseq Figure 3) and by Metformin (vs. vehicle) in LF-IPF (scRNAseq Figure 2) and in WI-38 (Figure 4). A) Venn diagram showing the indicated comparison (left) and **B)** the heatmap of the identified 67 genes common to the 3 comparisons. The log2 ratio for each condition is indicated in the table. The intensity of the red is directly proportional to the expression level.

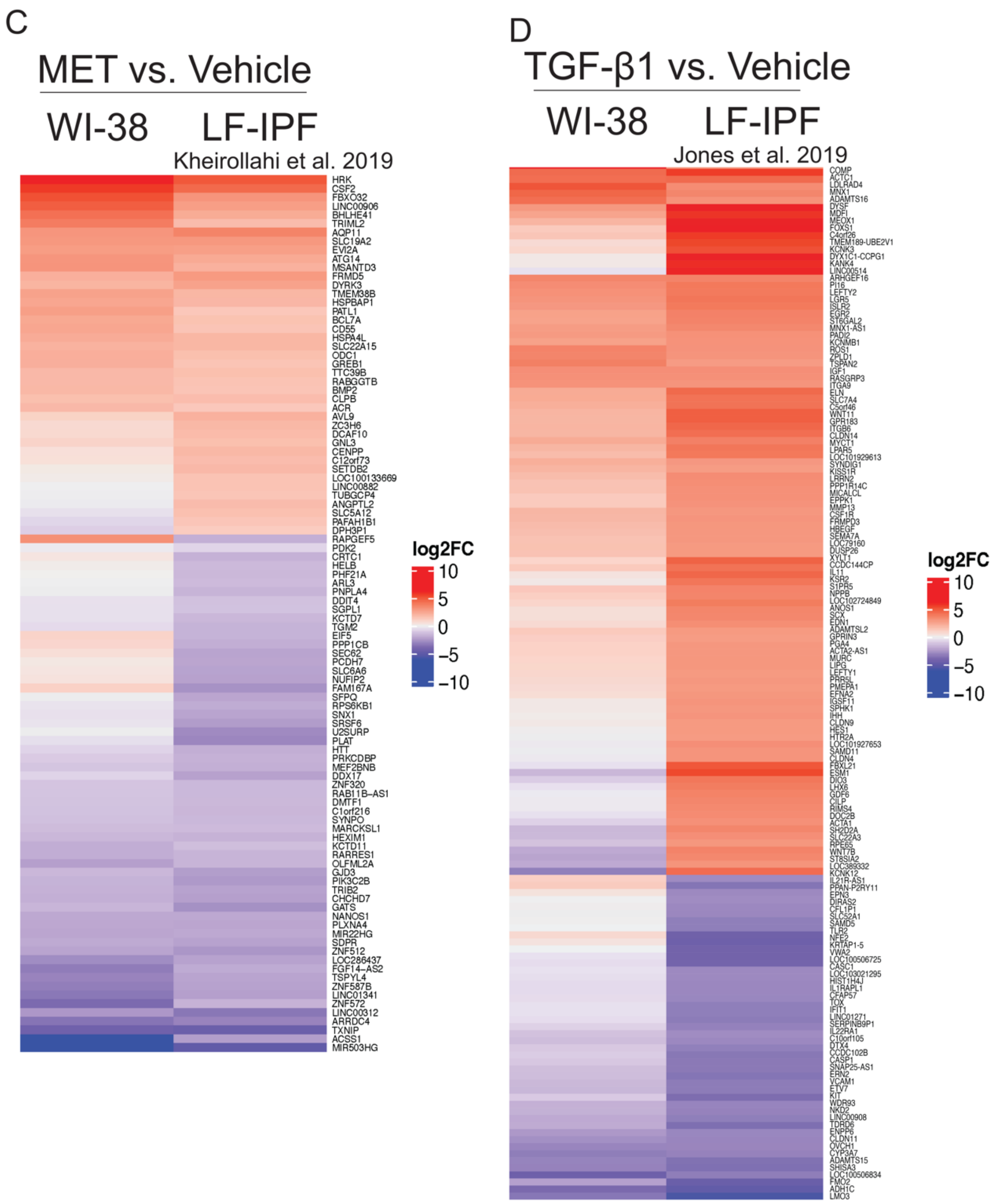

Figure S3: High magnification of the heatmap shown in Figure 4C and D

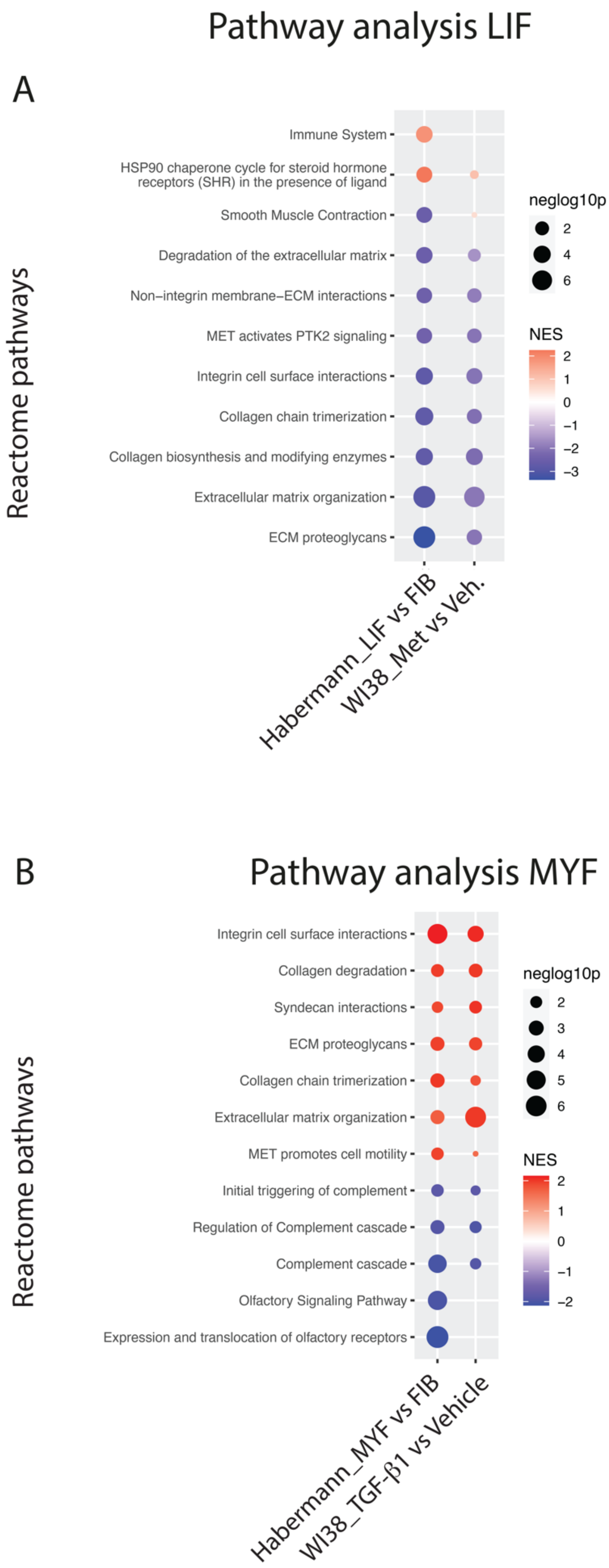

Figure S4: Pathway analysis for LIF and MYF using the Habermann dataset and WI-38 cells. **A)** Pathway analysis of the LIF using the Habermann *PLIN2*+ FIB vs FIB and WI-38 Met vs. Vehicle. **B)** Pathway analysis of the MYF using the Habermann MYF vs FIB and WI-38 TGF-β1 vs. Vehicle.

A

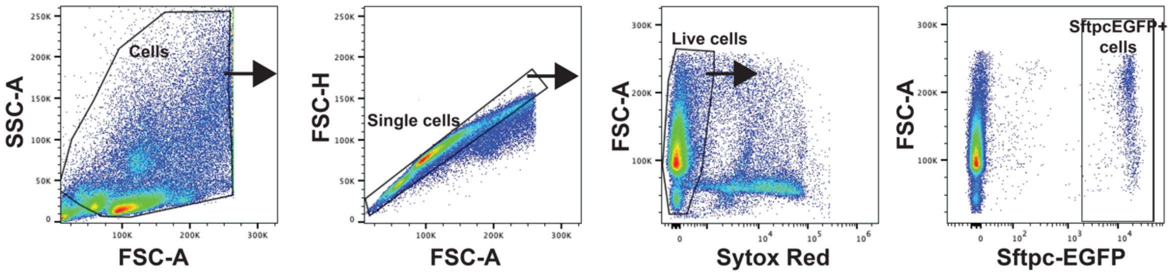

B

WI-38	differentiated cell name	first treatment	second treatment	endpoint
FIB	MYF1	TGF-b1	-	4 days
FIB	LIF1	Metformin	-	4 days
FIB	LIF2	TGF-b1	Metformin	4 days+4days
FIB	MYF2	TGF-b1	vehicle	4 days+4days
FIB	LIF-1 like	vehicle	Metformin	4 days+4days
FIB	MYF3	Metformin	TGF-b1	4 days+4days
FIB	LIF3	Metformin	vehicle	4 days+4days
FIB	MYF1-like	vehicle	TGF-b1	4 days+4days

Figure S5: A) Gating strategy for the isolation of GFP positive cells from adult Sftpc^{GFP+} mouse lungs. **B)** Abbreviation used for the different culture conditions

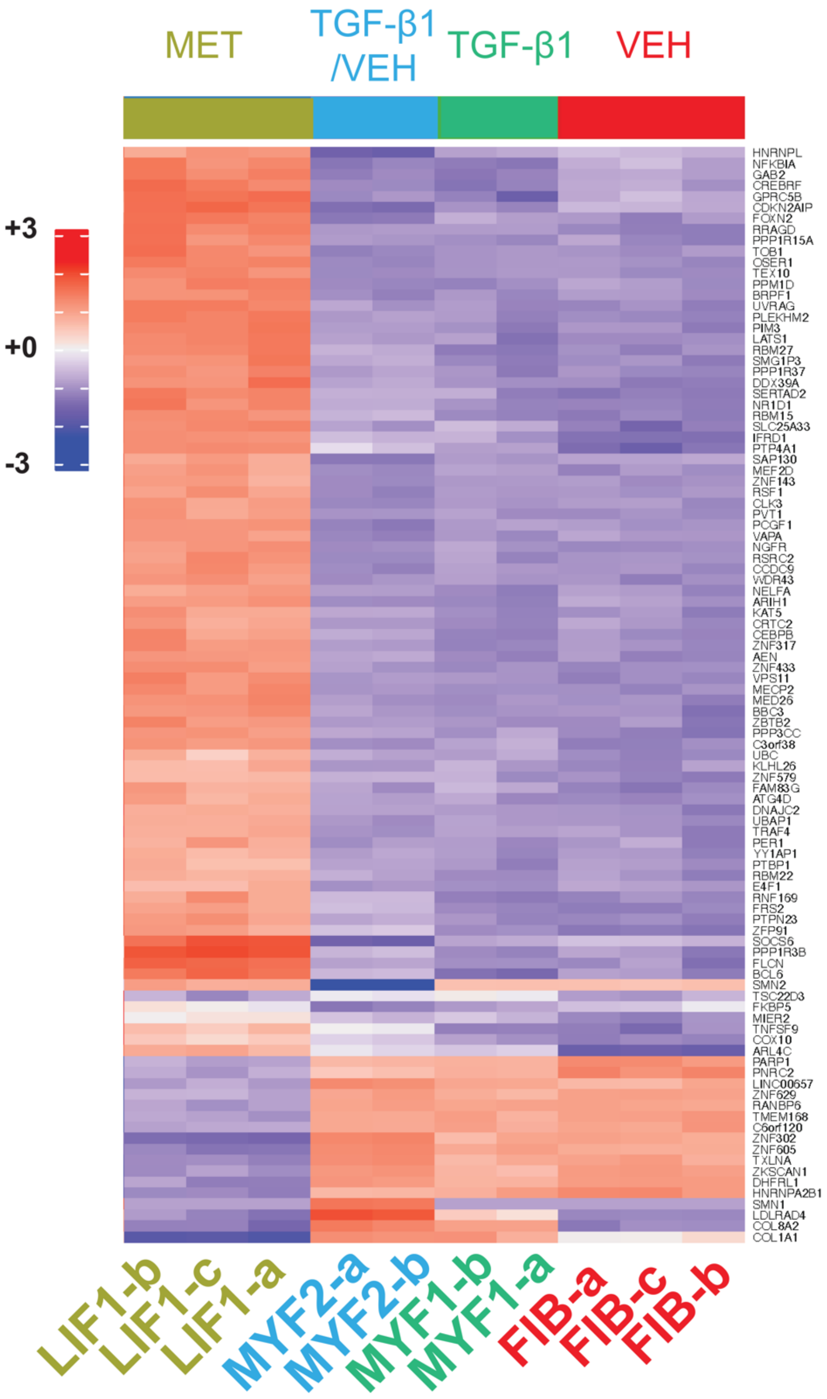

Figure S6: High magnification of the heatmap in Figure 7F
For LIF1 n = 3, MYF1 n = 2, MYF2 n = 2, FIB n = 3.
